# Overcoming a Conceptual Limitation of Industrial ε‐Caprolactone Production *via* Chemoenzymatic Synthesis in Organic Medium

**DOI:** 10.1002/cssc.202400073

**Published:** 2024-09-24

**Authors:** Laura Maria Bernhard, Harald Gröger

**Affiliations:** ^1^ Chair of Industrial Organic Chemistry and Biotechnology Faculty of Chemistry Bielefeld University Universitätsstr. 25 33615 Bielefeld Germany

**Keywords:** biocatalysis, ε-caprolactone, chemoenzymatic reaction, industrial chemistry, organic media

## Abstract

The multi‐10.000 tons scale manufactured chemical ε‐caprolactone attracts high industrial interest due to its favorable biodegradability properties. However, besides being of petrochemical origin yet, its production has a conceptual limitation that is the difficult extraction of this highly water‐soluble monomer from the water phase resulting from the aqueous solution of H_2_O_2_ applied as reagent. In this contribution, we report a chemoenzymatic cascade starting from bio‐based phenol, which makes use of O_2_ instead of H_2_O_2_ and runs in pure organic medium, thus requiring only simply decantation and distillation as work‐up. In a first step, phenol is hydrogenated quantitatively to cyclohexanol under solvent‐free conditions with a Ru‐catalyst. After simple removal of the heterogenous catalyst, cyclohexanol is converted to ε‐caprolactone in a biocatalytic double oxidation with very high yields just requiring O_2_ as reagent. This biocatalytic process proceeds in pure organic medium, thus avoiding tedious extraction to isolate the highly water‐soluble ε‐caprolactone and enabling a substantially simplified work‐up by only centrifugal separation of lyophilized whole cells and solvent removal. This oxidation is accomplished using a tailor‐made recombinant whole‐cell catalyst containing an alcohol dehydrogenase and a cyclohexanone monooxygenase mutant.

## Graphical abstract

A chemoenzymatic conversion of bio‐based phenol to the polymer building block ε‐caprolactone is achieved by an initial hydrogenation under solvent‐free conditions and subsequent biotransformation in organic media using lyophilized whole cells containing an alcohol dehydrogenase and a cyclohexanone monooxygenase, resulting in excellent conversion and simple isolation of ε‐caprolactone on preparative scale.

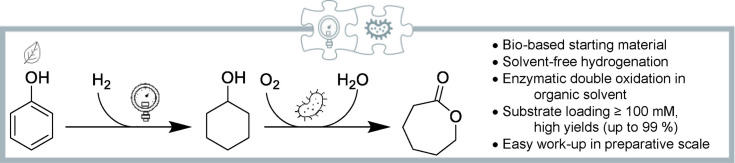

## Introduction

**Figure 1:**
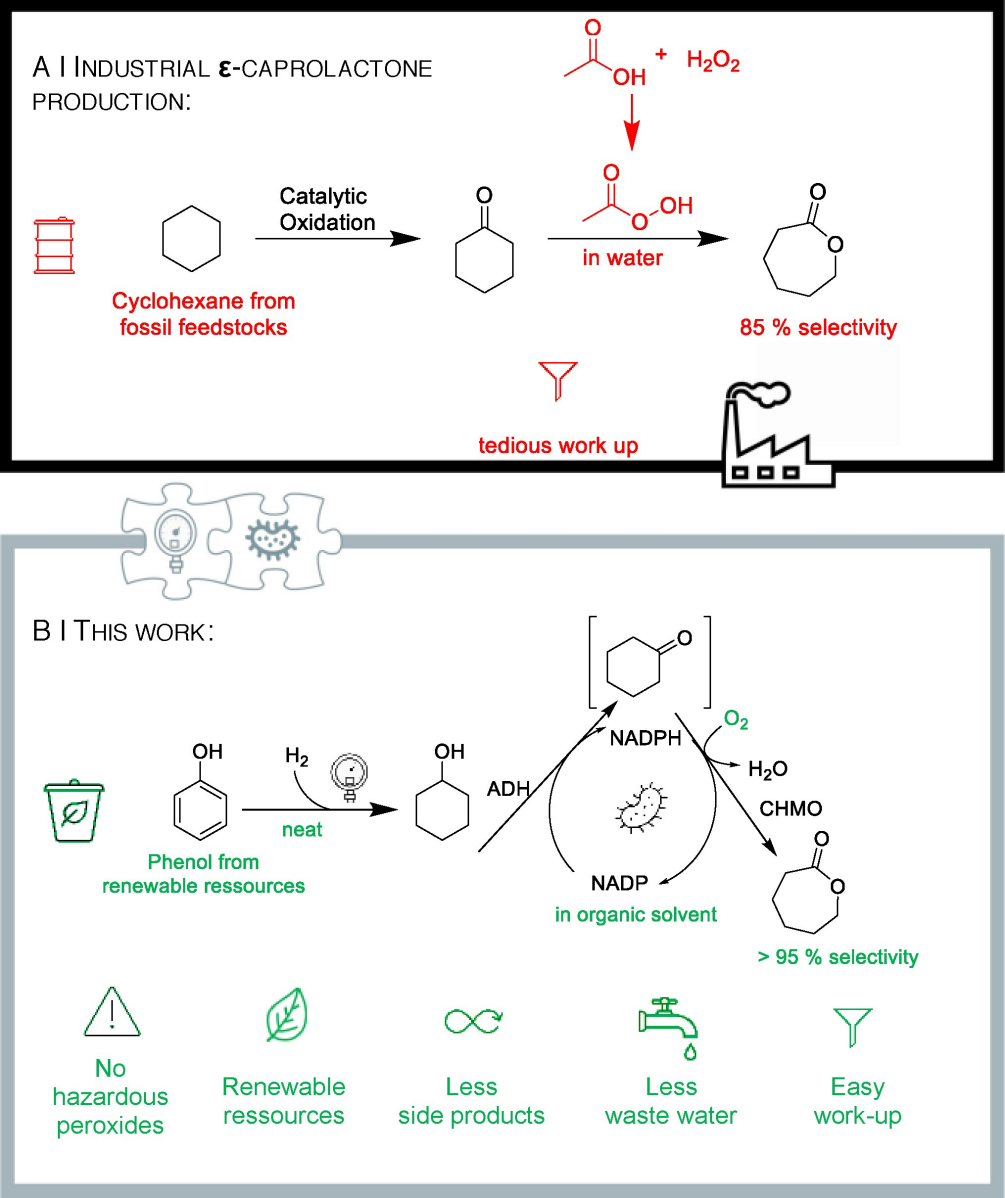
Industrial process for ε‐caprolactone production (**A**)^2^ *versus* the chemoenzymatic synthetic concept presented in this study (**B**).

How to access bio‐based & biodegradable polymers? The industrial demand for exactly such types of plastics is dramatically increasing, while at the same time the requirement to replace fossil feedstock becomes more and more urgent in general. One of such (very few!) large‐scale manufactured polymers fulfilling the prerequisite of being highly biodegradable^[1]^ is poly‐ε‐caprolactone, which explains the recent increasing interest in the monomer ε‐caprolactone being produced at an amount of multi‐10.000 tons scale.^[2]^ In spite of industrial interest in ε‐caprolactone, there are two major limitations. First, the raw material basis is still of fossil origin since the petrochemical cyclohexane serves as starting material in a cascade consisting of oxidation to “KA‐oil” (a mixture of cyclohexanol and cyclohexanone) and subsequent Baeyer‐Villiger oxidation of cyclohexanone to ε‐caprolactone. Second, this final oxidation step, which is carried out with aqueous hydrogen peroxide solution or peracetic acid, is just showing an 85 % selectivity (and, thus, substantial by‐product formation) and demands an (extractive or distillative) isolation of ε‐caprolactone, which is hampered by the unfavored high water‐solubility of ε‐caprolactone being in a multi‐hundred gram per liter range and results in disadvantageous extraction unit operation steps (Figure 1 A).[^2^, ^3^, ^4^, ^5^, ^6^, ^7^, ^8^]

About a decade ago we demonstrated a novel conceptual approach towards ε‐caprolactone, pioneering the first example of a pure biocatalytic approach for its formation just being based on molecular oxygen, directly used as air, as the sole reagent (and, thus, without the need for any co‐substrate for *in situ‐*cofactor recycling) when starting from cyclohexanol.^[9]^ This double oxidation approach proceeds in water and enables the access to ε‐caprolactone by oxidizing cyclohexanol to cyclohexanone in the presence of an alcohol dehydrogenase, followed by a Baeyer‐Villiger oxidation catalyzed by a monooxygenase. Since the cofactor‐recycling is done in a self‐sufficient mode, no external substrates are needed. Related concepts have been also developed and later this concept has been used and, in part, further modified by various groups, who also integrated this process in further cascades for preparing polymer building blocks or even oligomers and polymers.[^10^, ^11^, ^12^, ^13^, ^14^, ^15^, ^16^, ^17^, ^18^, ^19^, ^20^, ^21^] Together with collaboration partners, we also succeeded more recently in a “back integration” of this process to a bio‐based raw material: in detail, initial hydrogenation of bio‐based phenol gives cyclohexanol, which is then converted to ε‐caprolactone.^[22]^ What makes this chemoenzymatic process interesting for industry is the recent large scale availability of bio‐based phenol by Borealis.^[23]^ The industrial interest in this concept for bio‐based ε‐caprolactone is underlined by a current funded research project with BYK‐Chemie (ALTANA) as a leading international producer of additives for coatings and plastics.^[24]^

From a conceptual point of view, however, in analogy to today′s industrial process, also this biocatalytic double oxidation currently shows a limitation, which is related to the high water‐solubility of ε‐caprolactone.^[3]^ Since the biocatalytic double oxidation is carried out in aqueous medium, tedious extraction steps of the highly water‐soluble ε‐caprolactone are needed afterwards. In addition, extraction of batch‐type biotransformations raises emulsion formation concerns. Addressing the goal to overcome this hurdle and to reduce the number of unit operation steps, we envisaged a process being fully done in organic medium, ideally (on long term) in pure cyclohexanol as organic substrate, thus avoiding extractive work‐up steps and reducing the number of unit operation steps dramatically by a product isolation being simply based on distillation of the product from the reaction mixture. Note that, by definition, this concept would only be possible for the biocatalytic double oxidation and not (!) for today′s chemical Baeyer‐Villiger oxidation as water cannot be avoided due to the use of aqueous H_2_O_2_ solution (instead of O_2_ as in our process). Accordingly, we became interested in designing and using a tailor‐made whole‐cell catalyst for direct use in such an organic medium, which enables an improved and much more simplified process from a conceptual point of view than today′s routes. The difficulty of running such biocatalytic processes with cofactor‐dependent redox enzymes in pure organic media is demonstrated in a recently published work by the Kara group, which was performed independently and in parallel to our work. Therein, the highest substrate concentration was 60 mM, resulting in a maximum product formation of 4.5 mM ε‐caprolactone.^[25]^ Thus, the highest conversion did not exceed 8 %, indicating a strong biocatalyst deactivation and illustrating the high challenge to realize such a process concept. In the following, we report our results in this field, combined with a proof‐of‐concept, showing that by tailoring the biocatalyst formulation and reaction medium such a process can be realized now at substrate loadings of up to 100 mM and with very high conversion of up to 99 % (Figure 1 B).

## Results and Discussion

In order to synthesize ε‐caprolactone (**4**) starting from bio‐based phenol (**1**), we first investigated the widely studied[^26^, ^27^] hydrogenation of phenol (**1**) in more detail. For this purpose, we chose a ruthenium‐based heterogeneous catalyst as it catalyzes the selective transformation of phenol (**1**) into cyclohexanol (**3**),^[27]^ and is more cost‐effective than rhodium. However, to the best of our knowledge, there are only a very few examples for the hydrogenation of phenol (**1**) with ruthenium catalysts under solvent‐free conditions.^[28]^ For our work, we selected the commercially available ruthenium on activated carbon catalyst Escat™ 4401. To achieve a solvent‐free hydrogenation of phenol (**1**) using this heterogeneous catalyst, we made use of the low melting point of phenol (**1**, 41 °C) and conducted the reaction at a slightly higher temperature. This allows seperation of the product cyclohexanol (**3**) from the heterogeneous catalyst by simple filtration as easy work‐up. In all experiments, bio‐based phenol (**1**) from Borealis was used and underwent hydrogenation without the use of any solvent in the presence of a low catalyst loading of 0.0023 mol% within 12 hours. The reaction temperature and hydrogen pressure were adjusted as per requirement. Achieving quantitative conversion necessitated the utilization of a minimum reaction temperature of 70 °C alongside a H_2_‐pressure of 130 bar (or alternatively 80 °C and 100 bar) (Table 1, entries 4 & 5).

**Table 1:** Hydrogenation‐screening of bio‐based phenol (**1**) without solvent using Ru/C (Escat™ 4401) as heterogeneous catalyst.^[a]^

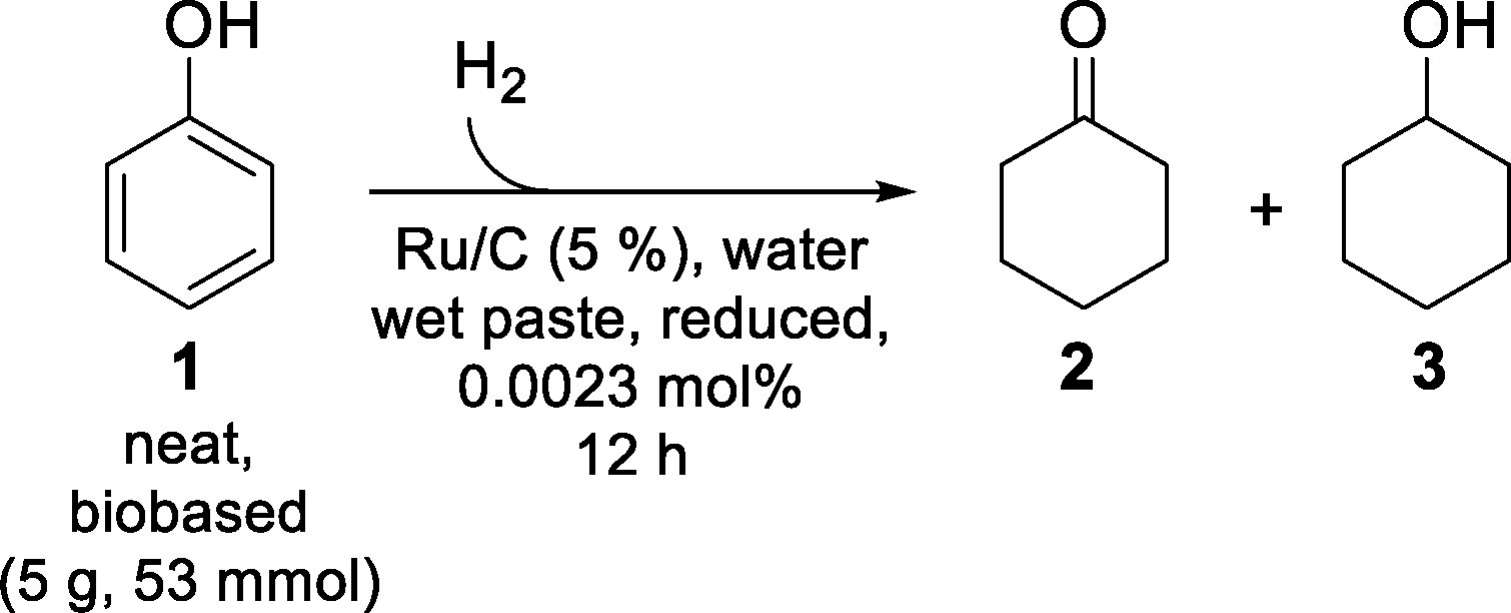					
entry	T/°C	p/bar	1/%^[b]^	2/%^[b]^	3/%^[b]^
1	60	120	5	0	95
2	60	130	5	1	94
3	70	120	1	0	99
4	70	130	0	0	>99
5	80	100	0	0	>99

[a] Hydrogenations were performed in high‐pressure autoclaves from Parr^®^. [b] Composition of the product mixture was calculated on the basis of the GC integrals.

After facile separation of the heterogeneous catalyst by filtration and without further purification, the obtained bio‐based cyclohexanol (**3**) was then directly utilized for the enzymatic double oxidation.

The biocatalytic synthesis of the polymer building block ε‐caprolactone (**4**), starting from cyclohexanol (**3**), is well‐documented in literature.^9,22^ However, the process is severely hindered by the high water‐solubility of ε‐caprolactone (**4**). This factor makes work‐up considerably difficult, which typically consists of tedious extraction steps and emulsion formation.[^9^, ^22^, ^25^] To overcome these hurdles, one conceptual option is to perform the biotransformation in a fully organic reaction medium, which ideally consists of an easily removable solvent or (as a long‐term perspective) only substrate and product as organic components. In such a desired organic system, the biocatalyst can be utilized in form of lyophilized whole cells, which are reactivated by adding a minimal amount of water to form a “hydration shell”.[^29^, ^30^] Such a process does not form a (visible) second aqueous phase in the reaction medium, thus enabling a work‐up *via* decantation only and consequently simplifying the work‐up procedure enormously.

To obtain ε‐caprolactone (**4**) from bio‐based phenol (**1**) *via* a chemoenzymatic reaction cascade combining an ADH and a CHMO, we constructed a suitable whole cell‐catalyst for the envisaged double‐oxidation from cyclohexanol (**3**) to ε‐caprolactone (**4**) in pure organic medium with cyclohexanone (**2**) as intermediate (Figure 1 B). A broad range of enzymes and plasmid combinations were selected and intensively investigated within a preliminary screening process, comprising ADHs from various organisms including those from *Thermoanaerobacter brockii subsp. finnii* (TbADH), *Thermoanaerobacter pseudethanolicus* (TeADH) and *Lactobacillus kefir* (LkADH) (Supporting Information, Figure S2) and cyclohexanone monooxygenases from *Acinetobacter* sp. NCIMB 9871 (AcinetoCHMO) as well as a mutant^[31]^ thereof. Various combinations thereof as well as further screening results are described in detail in the Supporting Information. Overexpressing both oxidoreductases, ADH and CHMO, within the same lyophilized cell enables an efficient transfer of the intermediate cyclohexanone (**2**) as well as the cofactors NADPH and NADP^+^ between the two enzymes, thus facilitating *in situ‐*cofactor regeneration.

The use of AcinetoCHMO and AcinetoCHMO‐mutant (mAcinetoCHMO)^[31]^ inserted into a pRSF‐Duet‐Vector, along with pACYC‐LkADH, demonstrated good co‐expression (Supporting Information, Figure S4). With the resulting recombinant *E. coli* whole cell‐catalysts in hand, high conversion rates were observed in initial analytical biotransformations of this double oxidation in aqueous media (Supporting Information, Figure S3). These two prioritized whole cell catalysts were further evaluated in organic reaction medium. Initial biotransformations were carried out again on an analytical scale utilizing these lyophilized whole cells at a cyclohexanol (**3**) concentration of 40 mM in cyclohexane and a buffer content of 10 % under oxygen atmosphere (Supporting Information, Figure S5). The results indicate that the monooxygenase mutant, which originally was found to be more stable against elevated temperatures,^[31]^ also turned out to be beneficial for the envisaged application in organic medium. While the whole cell‐catalyst based on the wild‐type monooxygenase only led to formation of 28 % ε‐caprolactone (**4**), a remarkably improved formation of product **4**, exceeding 80 %, was found when using the whole cell‐catalyst with the overexpressed mutant in organic media (82 % ε‐caprolactone (**4**) formation).

Once the best whole cell‐catalyst with promising performance in organic medium was identified, we investigated the impact of the type of organic solvent by testing various solvents ranging from highly hydrophobic (e. g., cyclohexane) to more polar solvents with lower logP‐values (e. g., ethyl acetate (Table 2)). In this study, also different solvents from renewable sources such as *n*‐butyl acetate, ethyl butyrate and a mixture of ethyl acetate, ethyl propionate and ethyl butyrate, readily available from short‐chain acids (being of waste stream origin), were evaluated. The study showed that in order to achieve optimal results a balance between hydrophilic and hydrophobic properties has to be ensured. Solvents with high hydrophobicity exhibit poor solubility of ε‐caprolactone (**4**). This leads to an accumulation of ε‐caprolactone (**4**) within the cells and their aqueous environment (hydration shell), potentially causing a severe enzyme inhibition. Conversely, solvents that possess excessive hydrophilicity display high water‐solubility (e. g., ethyl acetate (logP=0.73)), which leads to deactivation of the enzymes within the whole cell‐catalyst, resulting in reduced turnover numbers. In contrast, as highly suitable organic solvents the esters isopropyl acetate (logP=1.02) and *n*‐butyl acetate (logP=1.78) have been identified. In *n*‐butyl acetate, approximately 90 % of cyclohexanol (**3**) (logP=1.23) was converted to ε‐caprolactone (**4**) (logP=0.32) in only 2 hours reaction time of the double oxidation in organic media (Table 2). Additionally, this solvent is attractive from industrial perspective since *n*‐butyl acetate is a commonly used solvent due to its comparatively high boiling and flash point in contrast to other solvents.

**Table 2:** Screening of various solvents for the double‐oxidation of cyclohexanol (**3**) to ε‐caprolactone (**4**) in organic media.

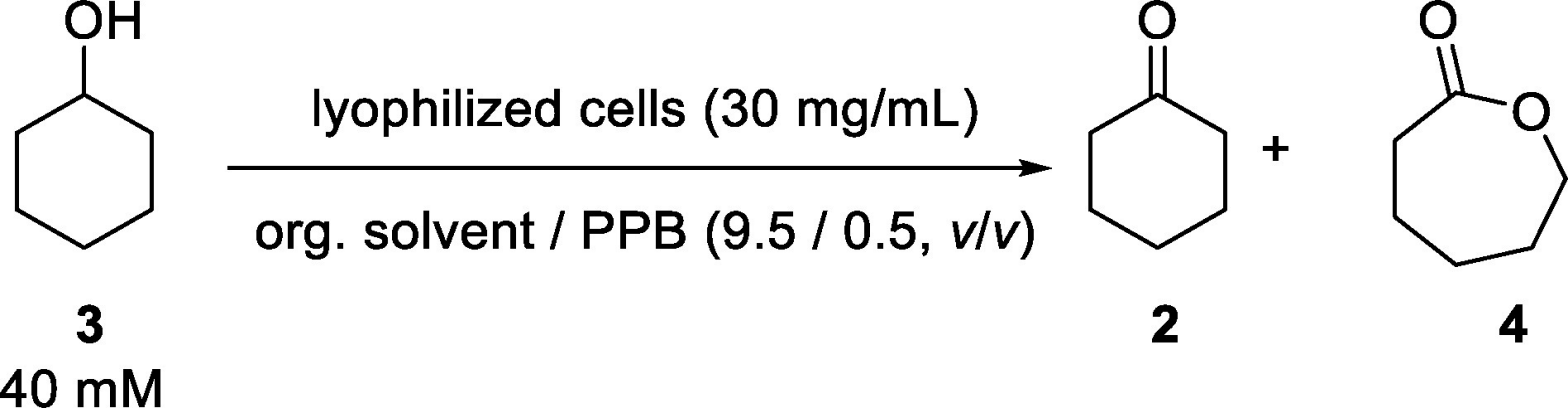				
entry	solvent	3/%^[a]^	2/%^[a]^	4/%^[a]^
1	cyclohexane	32	12	56
2	diethyl carbonate	88	8	4
3	methyl *tert*‐butyl ether	37	6	57
4	2,4‐dimethyl‐3‐pentanone	42	48	10
5	ethyl acetate	90	7	3
6	isopropyl acetate	25	4	71
7	*n*‐butyl acetate	10	3	86
8	ethyl butyrate	32	4	64
9	ethyl propionate	100	0	0
10	mixture of esters^[b]^	91	4	5
11	4‐methyl‐2‐pentanone^[c]^	36	62	2

[a] Percentages were determined *via* GC. Reaction conditions: 40 mM cyclohexanol (**3**) in different solvents and PPB (100 mM, pH 7, 9.5/0.5, *v*/*v*) and lyophilized whole‐cell catalyst loading of 30 mg mL^−1^; covered with oxygen and shaken at 25 °C and 850 rpm for 2 h. Lyophilized cells contain *E. coli* BL21(DE3) pRSF‐mAcinetoCHMO & pACYC‐LkADH. [b] Mixture of ethyl acetate, ethyl propionate and ethyl butyrate (1/1/1, *v*/*v*/*v*). [c] Oxidation of 4‐methyl‐2‐pentanone to the corresponding ester cannot be excluded based on this initial experiment.

**Figure 2:**
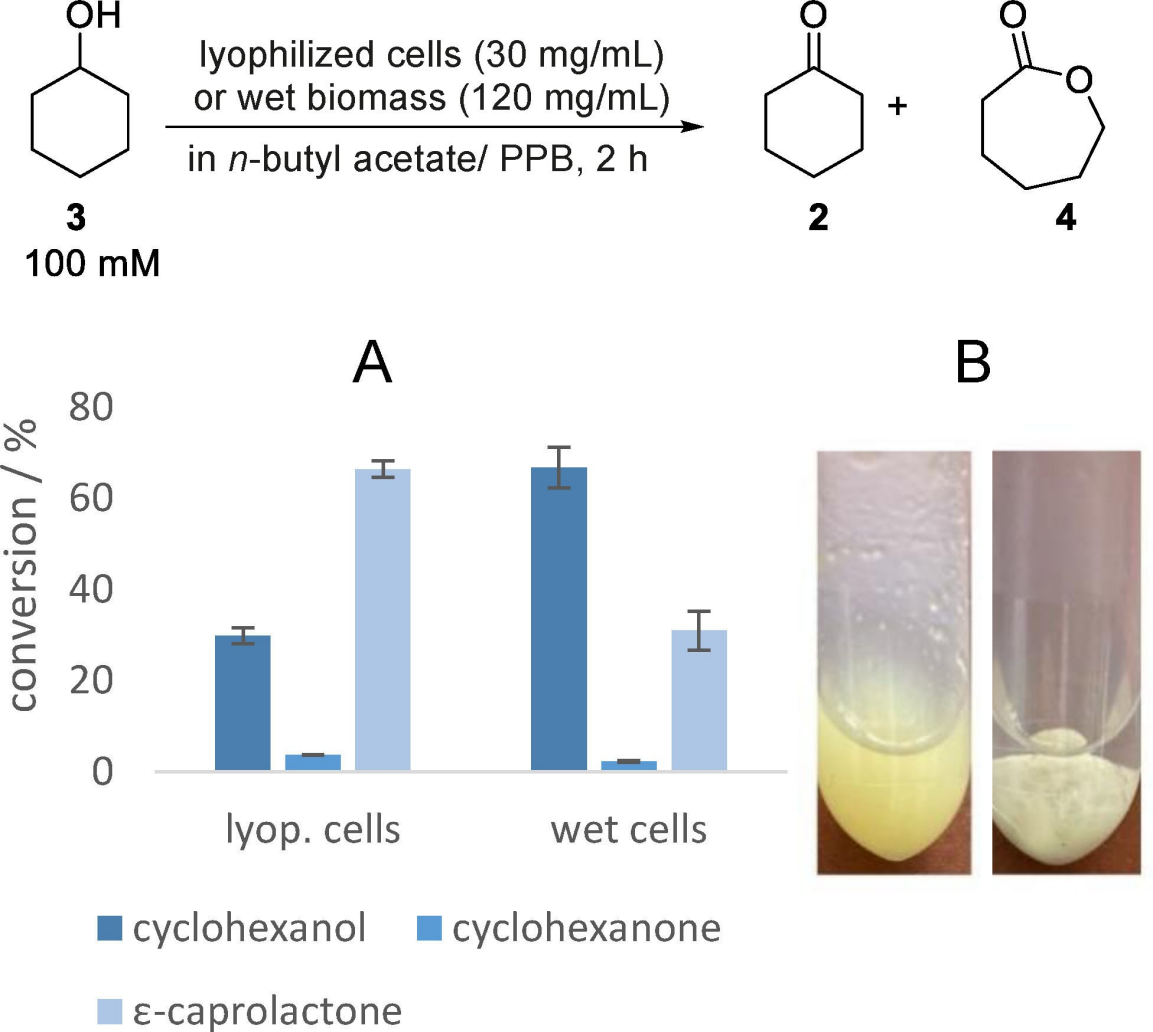
**A**: Analytic double oxidation of cyclohexanol (**3**) to ε‐caprolactone (**4**) (with cyclohexanone (**3**) as intermediate) in organic media. Reaction conditions: 100 mM cyclohexanol (**3**) in *n*‐butyl acetate and PPB (100 mM, pH 7, 9/1, *v*/*v* (lyop. cells) or 90/1, *v*/*v* (wet biomass)) and lyophilized whole‐cell catalyst with a loading of 30 mg mL^−1^ or whole‐cell catalyst as wet biomass with a loading of 120 mg mL^−1^ (*E. coli* BL21(DE3) pRSF‐mAcinetoCHMO & pACYC‐LkADH); covered with oxygen and shaken at 25 °C and 850 rpm. Conversions were determined *via* GC. **B**: Photos of the reactions; left: Reaction using lyophilized cells; right: Reaction using wet biomass.

A key prerequisite, however, is to identify a whole cell‐formulation, which enables both, high activity, and high stability in organic medium even under elevated substrate concentration. The importance of this task as well as the opportunity for a simple fine‐tuning of whole cell‐catalysts in order to make them “fit” for transformations in organic medium is mostly underestimated and widely unknown. In the following, we will show that the performance depends strongly on exactly such a fine‐tuning of the whole cell‐catalysts and that whole cell‐catalysts leading to unsatisfactory results gave high improvement by bringing them in the right formulation. In detail, we will demonstrate the superior suitability of lyophilized cells for application in pure organic medium compared to the “classic” form of wet biomass by conducting an experiment to convert cyclohexanol (**3**) (100 mM) in *n*‐butyl acetate using the enzymatic double oxidation technique with either lyophilized cells or wet biomass containing the same whole cell catalyst. In the experiment with lyophilized cells, 10 % aqueous buffer was added (without formation of a visible second aqueous phase) to reactivate the cells. For the experiment using wet biomass, due to its higher water content, four times the amount of lyophilized biomass was added to have a comparable overall biomass content, and also the overall amount of buffer was adjusted to obtain a comparable total amount of buffer in both experiments. Finally, we conducted identical reactions twice, once using lyophilized biomass and once using wet biomass. From the obtained results, which are shown in Figure 2, it is apparent that using lyophilized cells is more efficient than utilizing wet biomass.

Initially, the reason for that (at the first glance surprising) difference of the catalytic performance remains uncertain, as both formulations of the whole cell‐catalysts have the same biological composition. Upon further examination, however, it becomes clear that the structure of the cell mass differs significantly. Starting with lyophilized cells, the cell mass is finely distributed in the (visually) pure organic medium, while the wet biomass forms a lump upon entering the organic media. We expect that these findings can be generalized and, thus, will be of general relevance for further whole cell‐transformations in pure organic media. With respect to further applications, it shows that with the same enzymes and even same whole cells, completely different catalytic performances are achieved in dependency on the formulation of the whole cells.

**Figure 3:**
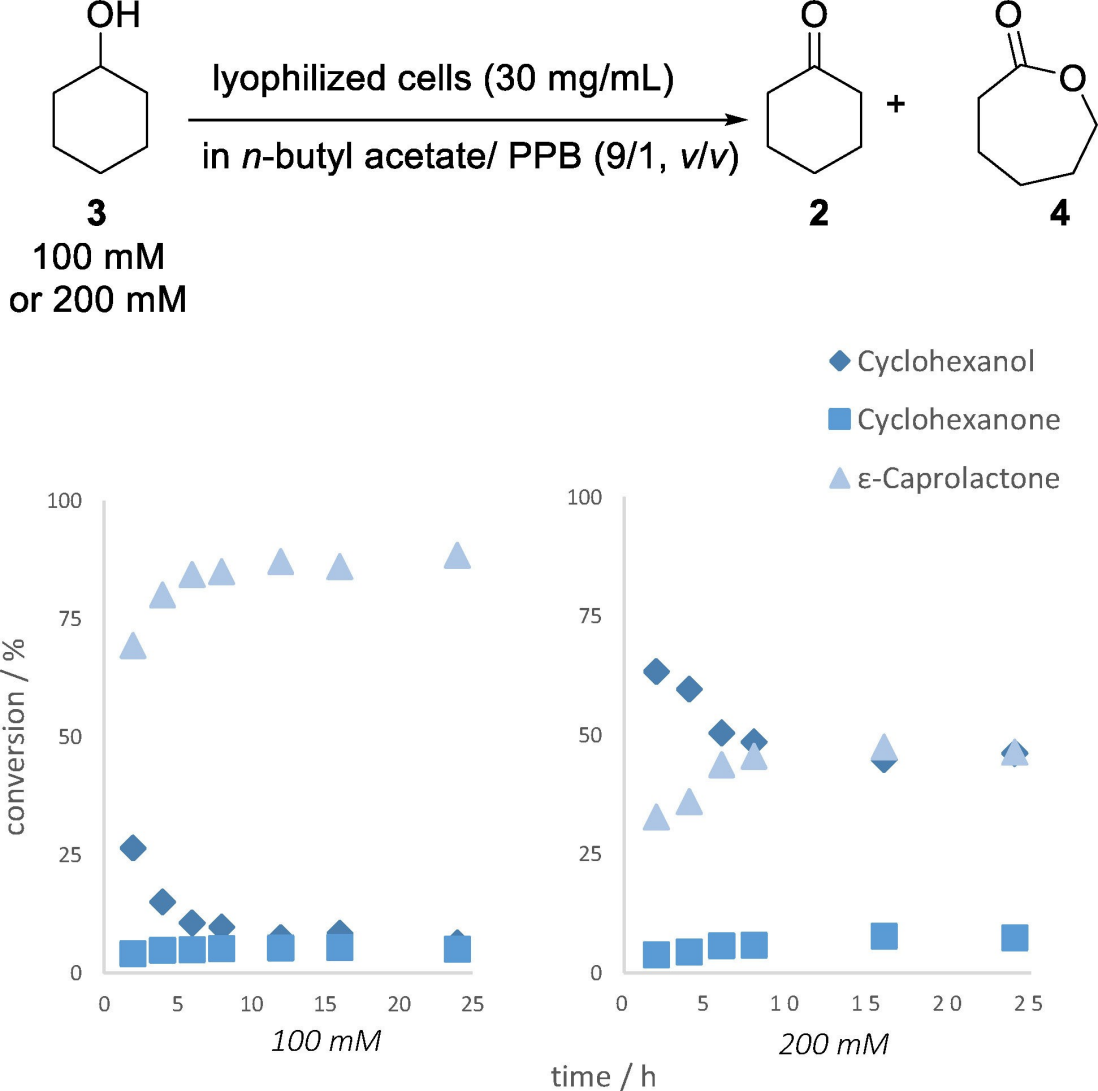
Kinetic measurements of the analytic double oxidation of cyclohexanol (**3**) to cyclohexanone (**2**) and ε‐caprolactone (**4**) in organic media for 24 h. Reaction conditions: 100 mM (left) respectively 200 mM (right) cyclohexanol (**3**) in *n*‐butyl acetate and PPB (100 mM, pH 7, 9/1, *v*/*v*) and lyophilized whole‐cell catalyst (*E. coli* BL21(DE3) pRSF‐mAcinetoCHMO & pACYC‐LkADH) with a loading of 30 mg mL^−1^; covered with oxygen and shaken at 25 °C and 850 rpm. Percentages were determined *via* GC.

Having the most suitable solvent for the enzymatic double oxidation in organic media in hand, we studied the conversion of different substrate loadings and the amount of buffer addition for whole cell‐catalyst fine tuning. First, the conversion of cyclohexanol (**3**) (40 mM) to ε‐caprolactone (**4**) in *n*‐butyl acetate was investigated. The 40 mM transformation was chosen as benchmark experiment, since the double oxidation in the range up to 60 mM was described with conversions being above 90 % in aqueous media, and at higher substrate loadings the overall conversion is known to drop significantly.[^9^, ^22^] After a reaction time of only 2 hours, conversions to ε‐caprolactone (**4**) in organic media were observed to be around 83 % and 97 % (containing 5 % or 10 % buffer additions, respectively). In addition, we were pleased to find that the conversion of cyclohexanol (**3**) at a further elevated substrate concentration of 80 mM in *n*‐butyl acetate (containing 10 % aqueous buffer) for the enzymatic double oxidation gave ε‐caprolactone (**4**) with a conversion above 90 % after only 6 hours. Furthermore, increasing the substrate concentration up to 100 mM utilizing our developed reaction system in organic medium still resulted in a high conversion of 87 % to ε‐caprolactone (**4**) after 12 hours (Figure 3). Subsequently, the substrate concentration was increased again to 200 mM in order to explore the limits of our system. The results show that at this increased substrate concentration the conversion drops and after 8 h the conversion to ε‐caprolactone (**4**) remaines at 46 % (Figure 3).

**Figure 4:**
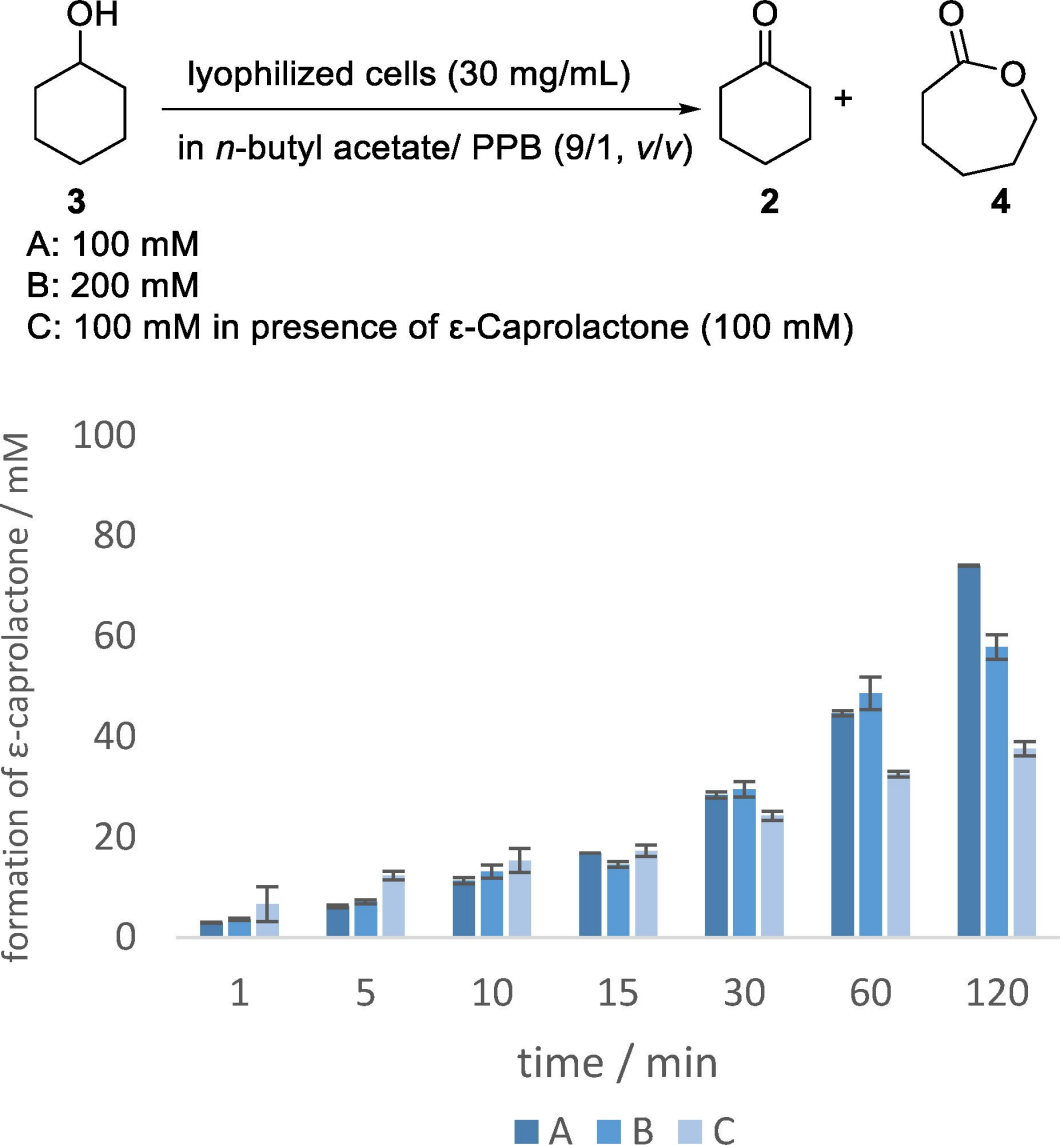
Kinetic measurements of the enzymatic double oxidation of cyclohexanol (**3**) towards ε‐caprolactone (**4**) in organic media. Reaction conditions: 100 mM (A, C) or 200 mM (B) of cyclohexanol (**3**) (C: in the presence of ε‐caprolactone (**4**) (100 mM)) in *n*‐butyl acetate and PPB (100 mM, pH 7, 9/1, *v*/*v*) and lyophilized whole‐cell catalyst (*E. coli* BL21(DE3) pRSF‐mAcinetoCHMO & pACYC‐LkADH) with a loading of 30 mg mL^−1^; covered with oxygen and shaken at 25 °C and 850 rpm. Molarities were calculated on the basis of the GC integrals.

As a next step, we became interested to gain insight why doubling the substrate concentration resulted in such a dramatic loss of conversion. First, kinetic data of initial reaction rates of the double oxidation towards ε‐caprolactone (**4**) in organic media were determined at substrate concentrations of 100 mM and 200 mM. In addition, an experiment of the conversion at 100 mM cyclohexanol (**3**) concentration in the presence of 100 mM ε‐caprolactone (**4**) was performed to investigate the effect of the initially increased ε‐caprolactone (**4**) concentration on the enzymatic double oxidation (Figure 4). Interestingly, these experiments show comparable initial reaction rates of the 100 and 200 mM conversions to ε‐caprolactone (**4**) . However, after a certain reaction progress there is a stage at which the reaction does not continue in case of the 200 mM double oxidation. Additionally, the initial reaction rates of the 100 mM conversion of cyclohexanol (**3**) in presence of 100 mM ε‐caprolactone (**4**) are comparable with the 100 mM conversion of cyclohexanol (**3**) for the first 15 minutes, but also in this experiment the adverse effect on the conversion is apparent after 30 minutes.

These results clearly show that the reaction progress is suppressed at higher concentrations of ε‐caprolactone (**4**). However, taking the achievements in directed evolution methods into account,^[32]^ which have been awarded Nobel Prize in Chemistry 2018,^[33]^ it can be expected that further improved BVMO mutants (as remaining “missing link” for using this technology on industrial scale) will be able to be discovered in the future, which do not show this extent of product inhibition by ε‐caprolactone (**4**) anymore and, thus, then enable this process at substrate concentrations beyond 200 mM.

In addition, we studied if other effects also could limit the reaction progress. For example, the natural occuring NADPH oxidase in *E. coli* had to be excluded as the cause of the stagnation of the reaction. In the case that the NADPH oxidase is highly active, the *in situ* regeneration system would not work. However, no NADPH consumption was detected in spectrophotometric activity assays, therefore such an undesired effect can be excluded (Supporting Information).

Another advantage of lyophilized cells is the increased stability of the overexpressed enzymes. It is important to mention that after 5 days of incubation at room temperature the lyophilized cells showed an average loss of turnover of about 5 % only (Supporting Information, Table S12).

Finally, we conducted the biocatalytic synthesis of ε‐caprolactone (**4**) at a preparative scale and applied different reactors for this purpose. Toward this end, we performed the reaction with cyclohexanol (**3**, 100 mM) on a 5 mL scale in a glass tube (stirring, 850 rpm) and in a centrifuge tube (shaking, 400 rpm) in organic reaction medium with *n*‐butyl acetate as prioritized organic solvent (and PPB (100 mM, pH 7), 9/1, *v*/*v*). Both reactions show comparable conversions of about 85 %. However, we observed that the cells in the centrifuge tube are still finely dispersed after shaking, whereas the lyophilized cells in the glass tube clump together after a short time and aggregate on the glass surface (Supporting Information, Figure S11). These results show that both, the reactor material and the type of mixing, are significant reaction parameters in biocatalysis. Accordingly, all following experiments were performed in centrifuge tubes.

**Scheme 1:**
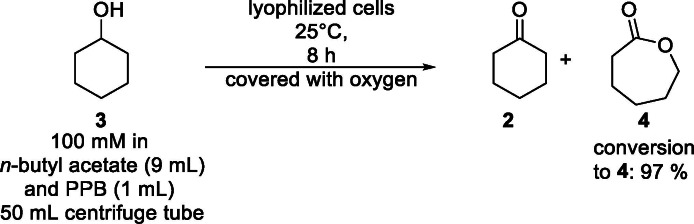
Semi‐preparative double oxidation of cyclohexanol (100 mM, 10 mL) to ε‐caprolactone (**4**) in organic media. Reaction conditions: 100 mM cyclohexanol in *n*‐butyl acetate and PPB (100 mM, pH 7, 9/1, *v*/*v*) and lyophilized whole‐cell catalyst (*E. coli* BL21(DE3) pRSF‐mAcinetoCHMO & pACYC‐LkADH) with a loading of 30 mg mL^−1^; covered with oxygen and shaken at 25 °C and 400 rpm in a centrifuge tube. Percentages were determined *via* GC.

In terms of further increasing the lab scale of the biotransformation, the reaction scale was doubled towards a semi‐preparative scale (10 mL, accomplished twice). Cyclohexanol (**3**) (100 mM, 10 mL) was transformed to ε‐caprolactone (**4**) in organic reaction medium system with *n*‐butyl acetate as organic media and PPB (9/1, *v*/*v*) in a centrifuge tube (shaking, 400 rpm) catalyzed by lyophilized cells (*E. coli* BL21(DE3) pRSF‐mAcinetoCHMO & pACYC‐LkADH; loading of 30 mg mL^−1^), leading to 97 % conversion towards ε‐caprolactone (**4**) for both reactions (Scheme 1). After removing cyclohexanol (**3**), cyclohexanone (**2**) and the solvent in *vacuo*, ε‐caprolactone (**4**) was isolated in an average yield of 85 %. The ^1^H‐NMR spectrum shows minor impurities in the product, e. g. polycaprolactone (2 %: for further details about the study of the impurity profile, see Supporting Information).

As the overall aim of our work was the synthesis of ε‐caprolactone (**4**) starting from bio‐based phenol (**1**), in the next experiment we performed the biotransformation in organic media in semi‐preparative scale (100 mM, 10 mL) starting with cyclohexanol (**3**) obtained from hydrogenation of bio‐based phenol (**1**) under solvent‐free conditions (Table 1, entry 5) using the identified optimized reaction conditions (Scheme 1). We were pleased to find a quantitative conversion of the biocatalytic double oxidation to ε‐caprolactone (**4**) within 8 h. After subsequent separation of the cells and removal of the solvent in *vacuo*, we obtained ε‐caprolactone (**4**) in 64 % yield (and with a similar impurity profile as before, containing, e. g., 2 % of polycaprolactone).

**Scheme 2:**
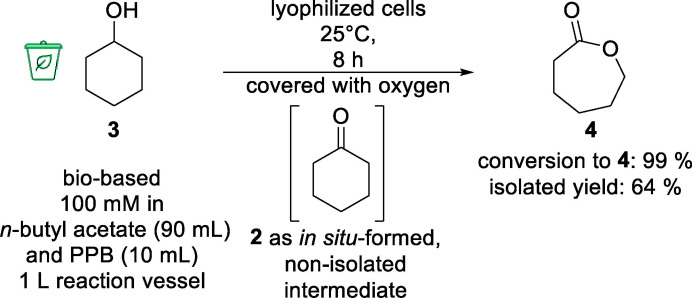
Preparative double oxidation of cyclohexanol (**3**) (100 mM, 100 mL) to ε‐caprolactone (**4**) in organic media. Reaction conditions: 100 mM cyclohexanol (**3**) in *n*‐butyl acetate and PPB (100 mM, pH 7, 9/1, *v*/*v*) and lyophilized whole‐cell catalyst (*E. coli* BL21(DE3) pRSF‐mAcinetoCHMO & pACYC‐LkADH) with a loading of 30 mg mL^−1^; covered with oxygen and shaken at 25 °C and 400 rpm in a 1 L plastic bottle as reaction vessel for 8 h. Purification *via* distillation.

To complete our work, we carried out the biotransformation on an elevated lab scale (100 mL) converting cyclohexanol (**3**, 100 mM), obtained from hydrogenation of bio‐based phenol (**1**) under solvent‐free conditions (Table 1, entry 5). Again, we performed the reaction in organic media, with *n*‐butyl acetate as the organic medium and PPB (9/1, *v*/*v*) in a 1 L reaction vessel (400 rpm shaking) and added lyophilized cells (*E. coli* BL21 (DE3) pRSF‐mAcinetoCHMO & pACYC‐LkADH) with a catalyst loading of 30 mg mL^−1^ (Scheme 2). We were pleased to find that an outstanding conversion of 99 % to ε‐caprolactone (**4**) from bio‐based cyclohexanol (**3**) (100 mM, corresponding to 10 g/L) was achieved. The work‐up consists of a simple purification *via* distillation and yielded ε‐caprolactone (**4**, 64 %) as a colorless liquid.

## Conclusions

In conclusion, a chemoenzymatic production process towards a bio‐based form of the biodegradable monomer ε‐caprolactone was developed, which also overcomes the current process limitation of tedious extraction to isolate this highly water‐soluble compound. Starting from industrially available bio‐based phenol from Borealis, hydrogenation with a heterogenous ruthenium‐based catalyst gives cyclohexanol in quantitative conversion under solvent free‐conditions. The following biocatalytic double oxidation of cyclohexanol was carried out in organic medium just requiring O_2_ as reagent and turned out to proceed efficiently with 99 % conversion to ε‐caprolactone when utilizing lyophilized cells as biocatalyst formulation. In contrast to today′s industrial route using aqueous H_2_O_2_, this chemoenzymatic process enables a simplified work‐up of the highly water‐soluble ε‐caprolactone, requiring only centrifugal separation and decantation from the lyophilized whole cells and solvent removal as work‐up steps. Thus, this contribution represents a good starting point for further investigation to obtain an industrially applicable process for this type of chemoenzymatic synthesis of ε‐caprolactone. Particular challenges to be further addressed are the achievement of substrate loadings of more than 2 M, which appear to be necessary in the field of such a bulk chemical, as well as an efficient recycling of the biocatalyst.

## Conflict of Interests

The authors declare no conflict of interest.

1

## Supporting information

As a service to our authors and readers, this journal provides supporting information supplied by the authors. Such materials are peer reviewed and may be re‐organized for online delivery, but are not copy‐edited or typeset. Technical support issues arising from supporting information (other than missing files) should be addressed to the authors.

Supporting Information

## Data Availability

The data that support the findings of this study are available in the supplementary material of this article.
