# Supplementary material for: Overcoming a Conceptual Limitation of Industrial ε‐Caprolactone Production via Chemoenzymatic Synthesis in Organic Medium
Source: ChemSusChem. 2024 Sep 24;17(22):e202400073. doi: 10.1002/cssc.202400073 (PMC11587681; doi:10.1002/cssc.202400073)
Supplement: Supplementary file 1 — Supporting Information [file CSSC-17-e202400073-s001.pdf]

ChemSusChem

Supporting Information

Overcoming a Conceptual Limitation of Industrial ε-Caprolactone Production *via* Chemoenzymatic Synthesis in Organic Medium

Laura Maria Bernhard and Harald Gröger*

Supporting Information

Overcoming a Conceptual Limitation of Industrial ε-Caprolactone Production *via* Chemoenzymatic Synthesis in Organic Medium

Laura M. Bernhard and Harald Gröger*

Table of content

1. Chemicals and standard working conditions 2

2. Analytics 3

3. Protein sequences, plasmids and expressions 5

4. Synthesis and Biotransformation 12

Hydrogenation of biobased phenol 12

Activity assay of ADHs 13

Analytical biotransformations to determine optimal vector system in aqueous media 14

Initial biotransformation in cyclohexane 16

Screening of different solvents for double-oxidation of cyclohexanol 16

Negative controls with empty vectors in organic media 17

Suitability of lyophilized cells vs. wet biomass 18

Double oxidation of cyclohexanol (40 respectively 80 mM) in *n*-butyl acetate 19

Double oxidation of cyclohexanol (100 respectively 200 mM) in *n*-butyl acetate 20

Biotransformation in *n*-butyl acetate - Initial reaction rates 21

Evaluation of the role of NADPH oxidase in lyophilized whole cell catalysts via spectrophotometric activity assays 23

Stability of the lyophilized cells in *n*-butyl acetate 24

Storage stability of lyophilized cells at room temperature 24

Biotransformation in organic media - 100 mM conversion on a 5 mL scale - glass vial vs. Falcon tube 25

Biotransformation in organic media - 100 mM conversion on a 10 mL scale 26

Biotransformation in organic media - 100 mM conversion of bio-based cyclohexanol on a 10 mL scale 27

Simulation of biotransformation on a semi-preparative scale without substrate 27

Biotransformation in organic media - 100 mM conversion of bio-based cyclohexanol on a 100 mL preparative scale 28

5. NMR-Spectra and GC-Chromatograms 29

6. References 32

1. Chemicals and standard working conditions

All chemicals were purchased in the highest purity and used without further processing.

β-Nicotinamide adenine-dinucleotide phosphate disodiumsalt (Carl Roth GmbH + Co. KG, Karlsruhe, Germany)

β-Nicotinamide adenine dinucleotide 2′-phosphate reduced tetrasodium salt (Carl Roth GmbH + Co. KG, Karlsruhe, Germany)

ε-Caprolactone (TCI Europe N.V., Zwijndrecht, Belgium)

Ampicillin sodium salt (Carl Roth GmbH + Co. KG, Karlsruhe, Germany)

n-Butyl acetate (Merck KGaA, Darmstadt, Germany)

Cyclohexane (VWR International GmbH, Darmstadt, Germany)

Cyclohexanol (Merck KGaA, Darmstadt, Germany)

Cyclohexanone (Carl Roth GmbH + Co. KG, Karlsruhe, Germany)

Chloramphenicol salt (Carl Roth GmbH + Co. KG, Karlsruhe, Germany)

Deuterated Chloroform (Eurisotop, St-Aubin Cedex, France)

Diethyl carbonate (Sigma-AldrichChemie GmbH, Steinheim, Germany)

2,4-Dimethyl-3-pentanone (TCI Europe N.V., Zwijndrecht, Belgium)

Dipotassium hydrogenphosphate (VWR International GmbH, Darmstadt, Germany)

Ethyl acetate (Fisher Scientific GmbH, Schwerte, Germany)

Ethyl propionate (Merck KGaA, Darmstadt, Germany)

Gylycerol (Carl Roth GmbH + Co. KG, Karlsruhe, Germany)

Isopropyl acetate (Merck KGaA, Darmstadt, Germany)

Isopropyl-β-D-thiogalactopyranoside (Carl Roth GmbH + Co. KG, Karlsruhe, Germany)

Hydrogen (Linde Gas, Bielefeld, Germany)

Kanamycin sulfate (Carl Roth GmbH + Co. KG, Karlsruhe, Germany)

LB-Medium (Carl Roth GmbH + Co. KG, Karlsruhe, Germany)

4-Methyl-2-pentanone (TCI Europe N.V., Zwijndrecht, Belgium)

Methyl *tert*-butyl ether (MTBE) (Evonik Oxeno GmbH & Co. KG, Essen, Germany)

Phenol, biobased (Borealis Polymers Oy, Porvoo, Finland)

Nitrogen (Linde Gas, Bielefeld, Germany)

Oxygen (Linde Gas, Bielefeld, Germany)

Potassium dihydrogenphosphate (CHEMSOLUTE® Laborchemikalien, Renningen, Germany)

Ruthenium, 5% on activated carbon, reduced, 50% water wet paste (Escat™ 4401) (Strem)

TB-Medium (Carl Roth GmbH + Co. KG, Karlsruhe, Germany)

All biotransformations were performed in an oxygen atmosphere. Hydrogenations were performed in a Parr Series 5000 Multiple Reactor System with enhanced safety precautions. Water was used as Milli-Q® water. Milli-Q® water was prepared using a Merck Milli-Q ultrapure water system.

All work with living organisms was performed under sterile conditions using the Mars Safety Class 2 sterile workbench from Dietmar Müller Labortechnik.

Solvents were removed on a rotary evaporator at 40 °C under reduced pressure.

2. Analytics

NMR data were collected using a Bruker Avance III 500 or Avance III 500 HD spectrometer at a frequency of 500 MHz (^{1}H) or 126 MHz (^{13}C) in chloroform-*d*. The spectra were referenced to the residual solvent peak in ppm (Chloroform-*d*: 7.26 ppm (s) for ^{1}H and 77.16 (t) for ^{13}C) and analyzed using MestReNova. Chemical shifts δ are given in ppm within 0.01 ppm. The multiplicity of ^{1}H signals has been abbreviated as follows: s = singulet, d = doublet, dd= doublet of doublet, t = triplet, q = quadruplet, m = multiplet or combinations thereof. Coupling constants (J) are given in Hz within 0.1 Hz.

Gas chromatographic analyses was performed in analogy to reference [22] on a GC2030 from Shimadzu Deutschland GmbH, Duisburg, Germany with a chiral BGB-174 column (BGB Analytik AG, Boeckten, Switzerland), with the autoinjector AOC-20i and a a flame ionisation detector (FID). The carrier gas was N_2. The injection volume was 1 µL with a split ratio of 1:10. Recorded spectra were analyzed using LABsolution software by Shimadzu. The settings are as follows: SPL1: 230 °C, pressure: 124.5 kPa, total flow: 25.2 mL min^{-1}, column flow: 2.11 mL min^{-1}, linear velocity: 46.9 cm s^{-1}, purge flow: 3.0 mL min-1, FID: 230 °C.[22] The temperature profile is shown in Table S1. The retention times of the analytes were as follows: cyclohexanol 3.3 min, cyclohexanone 3.7 min, phenol 4.4 min, ε-caprolactone 5.9 min. The signal intensities of various substances were considered by a calibration line and were adjusted for the extraction's dilution factor.

Table S1: Temperature profile of the GC analysis.

Rate / °C min^{-1}	Temperature / °C	Hold time / min
-	70	1
40	180	0
2	190	0
40	200	0

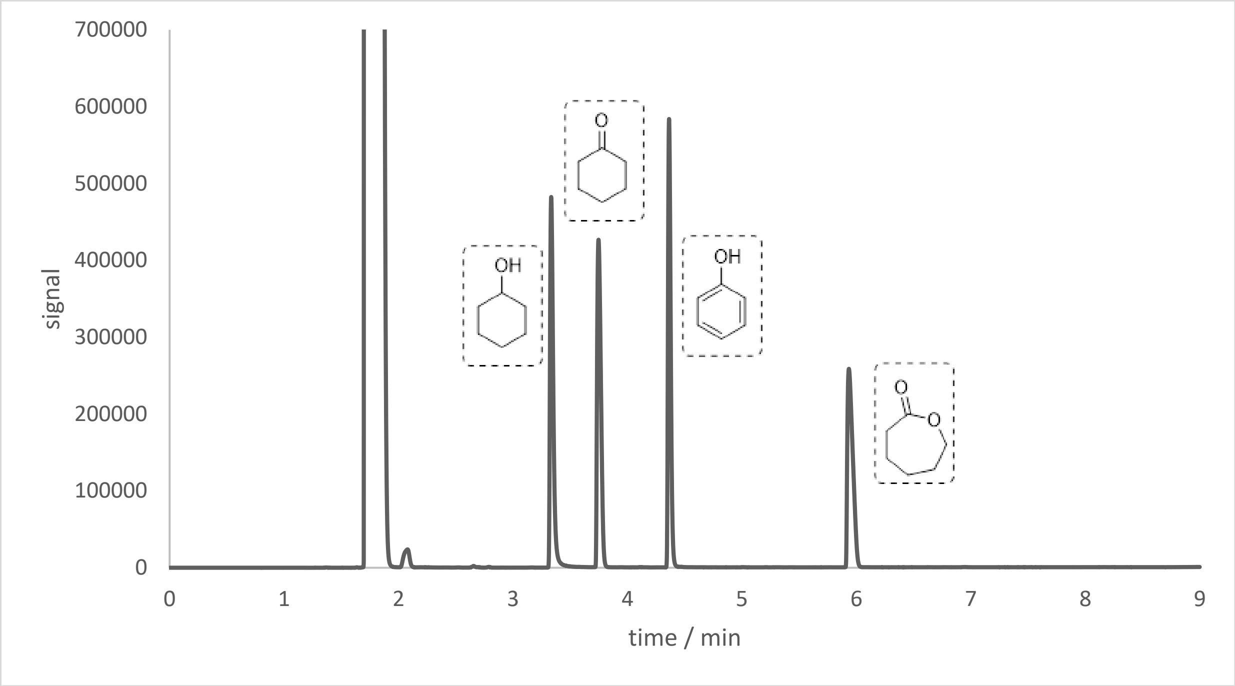

Figure S1: Exemplary GC spectrum of all components cyclohexanol **3**, cyclohexanone **2**, phenol **1** and ε-caprolactone **4**.

3. Protein sequences, plasmids and expressions

Alcohol-Dehydrogenase *from Thermoanaerobacter brockii subsp. finnii* Ako-1 (ATCC 43586 = DSM 3389) without Tag (Accession number: GenBank: X64841.1) Base sequence (not codon-optimized for *E. coli*): TbADH

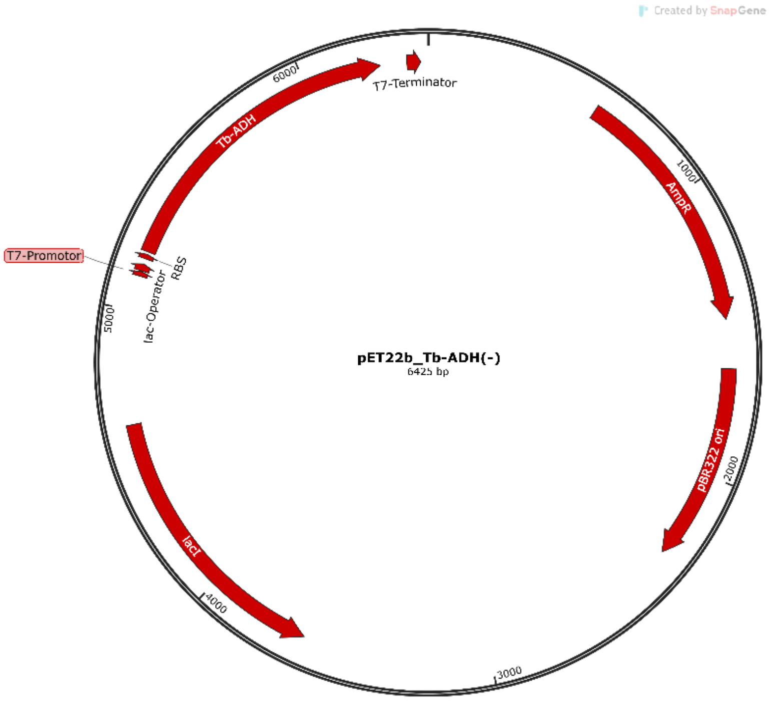

Base sequence (1059 bp):

ATGAAAGGTTTTGCAATGCTCAGTATCGGTAAAGTTGGCTGGATTGAGAAGGAAAAGCCTGCTCCTGGCCCAT
TTGATGCTATTGTAAGACCTCTAGCTGTGGCCCCTTGCACTTCGGACATTCATACCGTTTTTGAAGGCGCCATT
GGCGAAAGACATAACATGATACTCGGTCACGAAGCTGTAGGTGAAGTAGTTGAAGTAGGTAGTGAGGTAAAAG
ATTTTAAACCTGGTGATCGCGTTGTTGTGCCAGCTATTACCCCTGATTGGCGGACCTCTGAAGTACAAAGAGG
ATATCACCAGCACTCCGGTGGAATGCTGGCAGGCTGGAAATTTTCGAATGTAAAAGATGGTGTTTTTGGTGAAT
TTTTTCATGTGAATGATGCTGATATGAATTTAGCACATCTGCCTAAAGAAATTCCATTGGAAGCTGCAGTTATGAT
TCCCGATATGATGACCACTGGTTTTCACGGAGCTGAACTGGCAGATATAGAATTAGGTGCGACGGTAGCAGTT
TTGGGTATTGGCCCAGTAGGTCTTATGGCAGTCGCTGGTGCCAAATTGCGTGGAGCCGGAAGAATTATTGCC
GTAGGCAGTAGACCAGTTTGTGTAGATGCTGCAAAATACTATGGAGCTACTGATATTGTAAACTATAAAGATGGT
CCTATCGAAAGTCAGATTATGAATCTAACTGAAGGCAAAGGTGTCGATGCTGCCATCATCGCTGGAGGAAATG
CTGACATTATGGCTACAGCAGTTAAGATTGTTAAACCTGGTGGCACCATCGCTAATGTAAATTATTTTGGCGAAG
GAGAGGTTTTGCCTGTTCCTCGTCTTGAATGGGGTTGCGGCATGGCTCATAAAACTATAAAAGGCGGGCTATG
CCCCGGTGGACGTCTAAGAATGGAAAGACTGATTGACCTTGTTTTTTATAAGCGTGTCGATCCTTCTAAGCTCG
TCACTCACGTTTTCCGGGGATTTGACAATATTGAAAAAGCCTTTATGTTGATGAAAGACAAACCAAAAGACCTA
ATCAAACCTGTTGTAATATTAGCATAA

Amino acid sequence (352):

MKGFAMLSIGKVGWIEKEKPAPGPFDAIVRPLAVAPCTSDIHTVFEGAIGERHNMILGHEAVGEVVEVGSEVKDFKP
GDRVVVPAITPDWRTSEVQRGYHQHSGGMLAGWKFSNVKDGVFGEFFHVNDADMNLAHLPKEIPLEAAVMIPDM
MTTGFHGAELADIELGATVAVLGIGPVGLMAVAGAKLRGAGRIIAVGSRPVCVDAAKYYGATDIVNYKDGPIESQIM

NLTEGKGVDAAIIAGGNADIMATAVKIVKPGGTIANVNYFGEGEVLPVPRLEWGCGMAHKTIKGGLCPGGRLRMER
LIDLVFYKRVDPSKLVTHVFRGFDNIEKAFMLMKDKPKDLIKPVVILA

Alcohol-Dehydrogenase from *Thermoanaerobacter pseudethanolicus* 39E (ATCC 33223) without Tag (Accession number: GenBank: ABY93890.1) Base sequence (not codon-optimized for *E. coli*): TeADH

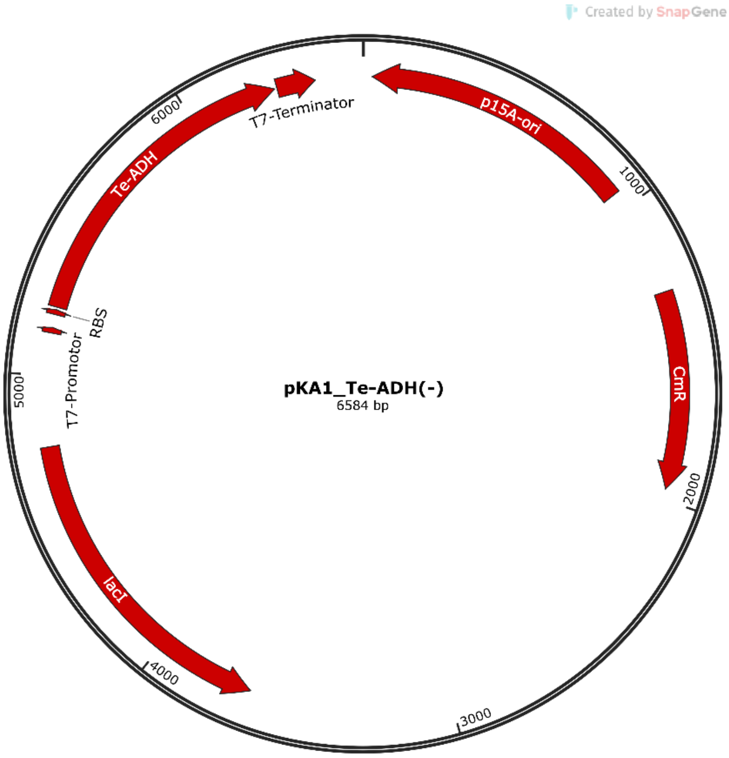

Base sequene (1062 bp):
ATGATGAAAGGTTTTGCAATGCTCAGTATCGGTAAAGTTGGCTGGATTGAGAAGGAAAAGCCTGCTCCTGGCC
CATTTGATGCTATTGTAAGACCTCTAGCTGTGGCCCCTTGCACTTCGGACATTCATACCGTTTTTGAAGGCGCC
ATTGGCGAAAGACATAACATGATACTCGGTCACGAAGCTGTAGGTGAAGTAGTTGGAGTAGGTAGTGAGGTAA
AAGATTTTAAACCTGGTGATCGCGTTGTTGTGCCAGCTATTACCCCTGATTGGCGGACCTCTGAAGTACAAAG
AGGATATCACCAGCACTCCGGTGGAATGCTGGCAGGCTGGAAATTTTCGAATGTAAAAGATGGTGTTTTTGGT
GAATTTTTTCATGTGAATGATGCTGATATGAATTTAGCACATCTGCCTAAAGAAATTCCATTGGAAGCTGCAGTT
ATGATTCCCGATATGATGACCACTGGTTTTCACGGAGCTGAACTGGCAGATATAGAATTAGGTGCGACGGTAG
CAGTTTTGGGTATTGGCCCAGTAGGTCTTATGGCAGTCGCTGGTGCCAAATTGCGTGGAGCCGGAAGAATTAT
TGCCGTAGGCAGTAGACCAGTTTGTGTAGATGCTGCAAAATACTATGGAGCTACTGATATTGTAAACTATAAAGA
TGGTCCTATCGAAAGTCAGATTATGAATCTAACTGAAGGCAAAGGTGTCGATGCTGCCATCATCGCTGGAGGA
AATGCTGACATTATGGCTACAGCAGTTAAGATTGTTAAACCTGGTGGCACCATCGCTAATGTAAATTATTTTGGC
GAAGGAGAGGTTTTGCCTGTTCCTCGTCTTGAATGGGGTTGCGGCATGGCTCATAAAACTATAAAAGGCGGG
CTATGCCCCGGTGGACGTCTAAGAATGGAAAGACTGATTGACCTTGTTTTTTATAAGCGTGTCGATCCTTCTAA
GCTCGTCACTCACGTTTTCCGGGGATTTGACAATATTGAAGAAGCCTTTATGTTGATGAAAGACAAACCAAAAG
ACCTAATCAAACCTGTTGTAATATTAGCATAA

Amino acid sequence (353):

MMKGFAMLSIGKVGWIEKEKPAPGPFDAIVRPLAVAPCTSDIHTVFEGAIGERHNMILGHEAVGEVVGVGSEVKDF
KPGDRVVVPAITPDWRTSEVQRGYHQHSGGMLAGWKFSNVKDGVFGEFFHVNDADMNLAHLPKEIPLEAAVMIP
DMMTTGFHGAELADIELGATVAVLGIGPVGLMAVAGAKLRGAGRIIAVGSRPVCVDAAKYYGATDIVNYKDGPIESQ
IMNLTEGKGVDAAIIAGGNADIMATAVKIVKPGGTIANVNYFGEGEVLPVPRLEWGCGMAHKTIKGGLCPGGRLRM
ERLIDLVFYKRVDPSKLVTHVFRGFDNIEEAFMLMKDKPKDLIKPVVILA

Alcohol-Dehydrogenase from *Lactobacillus kefir* DSM 20587 (JCM 5818) on pET21a without Tag (Accession number: GenBank: AY267012) Base sequence (not codon-optimized for *E. coli*): pET21a-LkADH

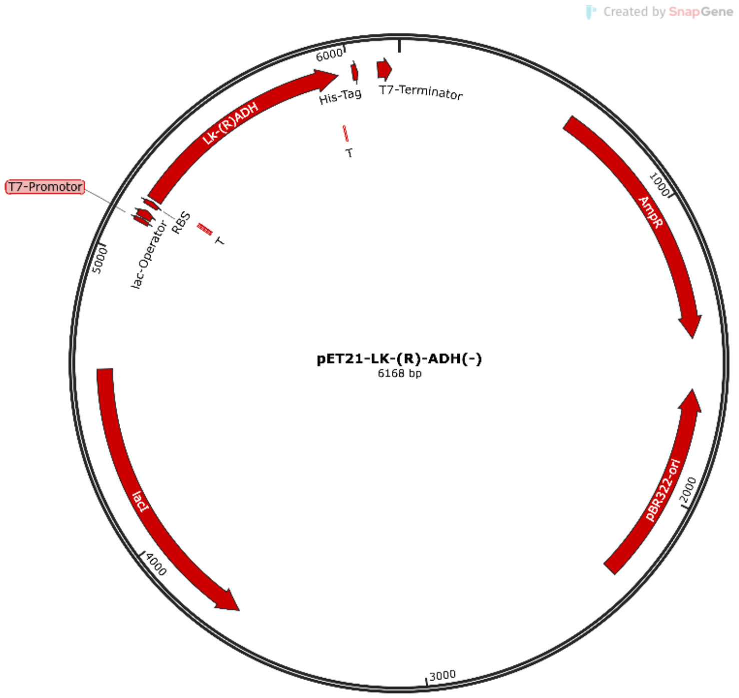

Base sequene (759 bp):

ATGACTGACCGTTTGAAAGGTAAGGTAGCAATTGTAACTGGCGGTACCTTGGGAATTGGCTTGGCAATCGCTG
ATAAGTTTGTTGAAGAAGGCGCAAAGGTTGTTATTACCGGCCGTCACGCTGATGTAGGTGAAAAAGCTGCCAA
ATCAATCGGCGGCACAGACGTTATCCGTTTTGTCCAACACGATGCTTCTGATGAAGCCGGCTGGACTAAGTTG
TTTGATACGACTGAAGAAGCATTTGGCCCAGTTACCACGGTTGTCAACAATGCCGGAATTGCGGTCAGCAAGA
GTGTTGAAGATACCACAACTGAAGAATGGCGCAAGCTGCTCTCAGTTAACTTGGATGGTGTCTTCTTCGGTAC
CCGTCTTGGAATCCAACGTATGAAGAATAAAGGACTCGGAGCATCAATCATCAATATGTCATCTATCGAAGGTTT
TGTTGGTGATCCAACTCTGGGTGCATACAACGCTTCAAAAGGTGCTGTCAGAATTATGTCTAAATCAGCTGCCT
TGGATTGCGCTTTGAAGGACTACGATGTTCGGGTTAACACTGTTCATCCAGGTTATATCAAGACACCATTGGTT
GACGATCTTGAAGGGGCAGAAGAAATGATGTCACAGCGGACCAAGACACCAATGGGTCATATCGGTGAACCT
AACGATATCGCTTGGATCTGTGTTTACCTGGCATCTGACGAATCTAAATTTGCCACTGGTGCAGAATTCGTTGT
CGACGGTGGCTACACTGCTCAATAG

Amino acid sequence (252):

MTDRLKGKVAIVTGGTLGIGLAIADKFVEEGAKVVITGRHADVGEKAAKSIGGTDVIRFVQHDASDEAGWTKLFDTT
EEAFGPVTTVVNNAGIAVSKSVEDTTTEEWRKLLSVNLDGVFFGTRLGIQRMKNKGLGASIINMSSIEGFVGDPTL
GAYNASKGAVRIMSKSAALDCALKDYDVRVNTVHPGYIKTPLVDDLEGAEEMMSQRTKTPMGHIGEPNDIAWICVY
LASDESKFATGAEFVVDGGYTAQ

Alcohol-Dehydrogenase from *Lactobacillus kefir* DSM 20587 (JCM 5818) on pACYCDuet without Tag (Accession number: GenBank: AY267012) Base sequence (not codon-optimized for *E. coli*): pACYC-LkADH

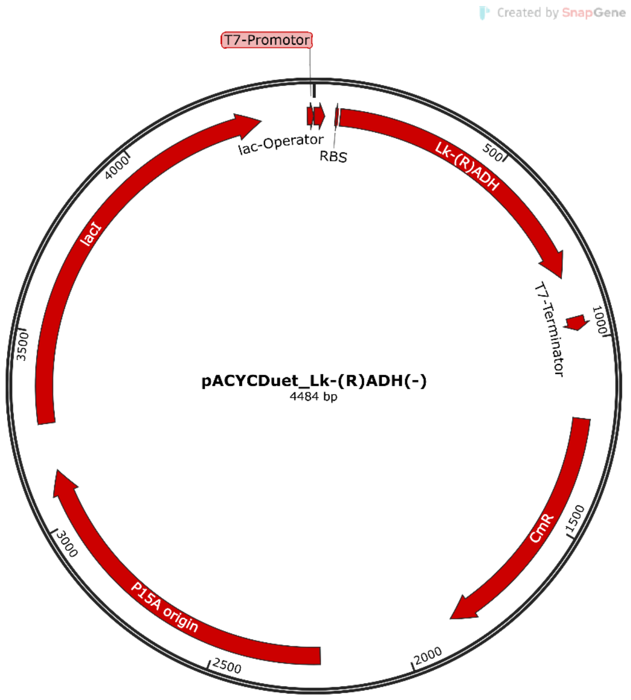

Base sequene (759 bp):

ATGACTGACCGTTTGAAAGGTAAGGTAGCAATTGTAACTGGCGGTACCTTGGGAATTGGCTTGGCAATCGCTG
ATAAGTTTGTTGAAGAAGGCGCAAAGGTTGTTATTACCGGCCGTCACGCTGATGTAGGTGAAAAAGCTGCCAA
ATCAATCGGCGGCACAGACGTTATCCGTTTTGTCCAACACGATGCTTCTGATGAAGCCGGCTGGACTAAGTTG
TTTGATACGACTGAAGAAGCATTTGGCCCAGTTACCACGGTTGTCAACAATGCCGGAATTGCGGTCAGCAAGA
GTGTTGAAGATACCACAACTGAAGAATGGCGCAAGCTGCTCTCAGTTAACTTGGATGGTGTCTTCTTCGGTAC
CCGTCTTGGAATCCAACGTATGAAGAATAAAGGACTCGGAGCATCAATCATCAATATGTCATCTATCGAAGGTTT
TGTTGGTGATCCAACTCTGGGTGCATACAACGCTTCAAAAGGTGCTGTCAGAATTATGTCTAAATCAGCTGCCT
TGGATTGCGCTTTGAAGGACTACGATGTTCGGGTTAACACTGTTCATCCAGGTTATATCAAGACACCATTGGTT
GACGATCTTGAAGGGGCAGAAGAAATGATGTCACAGCGGACCAAGACACCAATGGGTCATATCGGTGAACCT
AACGATATCGCTTGGATCTGTGTTTACCTGGCATCTGACGAATCTAAATTTGCCACTGGTGCAGAATTCGTTGT
CGACGGTGGCTACACTGCTCAATAG

Amino acid sequence (252):

MTDRLKGKVAIVTGGTLGIGLAIADKFVEEGAKVVITGRHADVGEKAAKSIGGTDVIRFVQHDASDEAGWTKLFDTT
EEAFGPVTTVVNNAGIAVSKSVEDTTTEEWRKLLSVNLDGVFFGTRLGIQRMKNKGLGASIINMSSIEGFVGDPTL
GAYNASKGAVRIMSKSAALDCALKDYDVRVNTVHPGYIKTPLVDDLEGAEEMMSQRTKTPMGHIGEPNDIAWICVY
LASDESKFATGAEFVVDGGYTAQ

Cyclohexanone monooxygenase from *Acinetobacter* sp. NCIMB 9871 (Acinetobacter johnsonii) on a pRSF_Duet with tag (N-His) (Accession number: GenBank: AB006902.2) Base sequence (codon-optimized for *E. coli*): pRSF-AcinetoCHMO

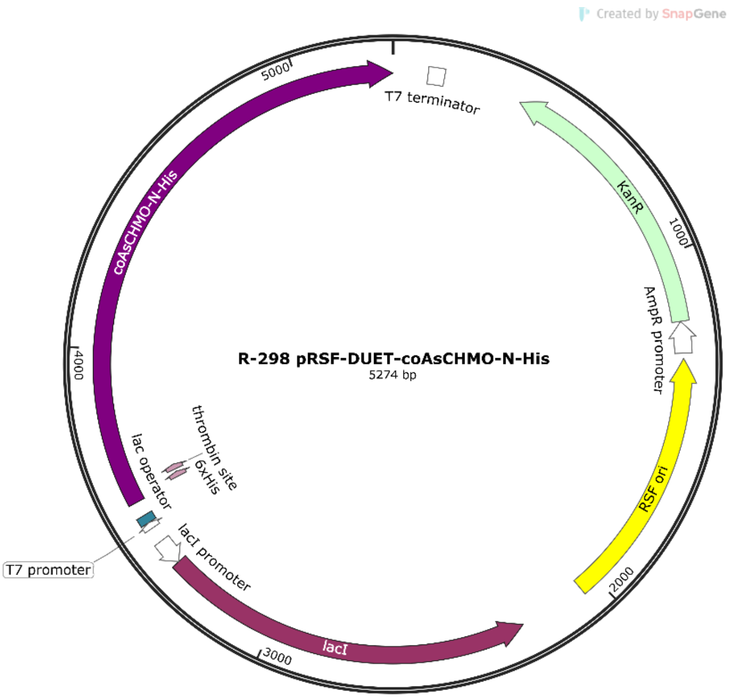

Base sequence (1734 bp)

ATGGGAAGTTCACATCACCATCACCACCACTCTAGCGGTCTTGTTCCGCGTGGTAGCCACATGGCGTCCATGA
CCGGCGGCCAGCAAATGGGCCGTGGTAGCATGTCTCAGAAAATGGATTTTGATGCCATCGTCATCGGTGGCG
GATTCGGCGGTCTGTACGCCGTGAAGAAGCTGCGTGACGAATTGGAGCTCAAAGTGCAGGCATTCGACAAA
GCGACTGACGTGGCGGGTACATGGTATTGGAATCGCTATCCGGGTGCCTTGACCGACACCGAAACCCATTTG
TACTGCTATAGCTGGGACAAGGAACTTCTGCAATCCCTGGAGATCAAGAAGAAGTATGTCCAAGGTCCGGATG
TGCGCAAATACCTGCAGCAAGTGGCCGAGAAGCACGATCTGAAAAAAAGCTATCAGTTCAATACCGCAGTTCA
ATCTGCTCATTACAATGAAGCTGACGCTCTGTGGGAAGTTACCACTGAATACGGCGACAAGTACACCGCTCGT
TTCCTGATTACGGCACTGGGTCTGCTCAGCGCACCGAATCTTCCGAACATTAAGGGTATTAACCAGTTCAAAG
GTGAATTGCACCACACCAGCCGTTGGCCGGACGACGTGTCGTTCGAGGGCAAGCGTGTTGGCGTGATCGGC
ACGGGTTCGACCGGTGTCCAGGTCATCACTGCGGTGGCGCCACTGGCAAAGCACTTGACCGTATTTCAAAGA
AGCGCTCAATACTCAGTTCCAATTGGCAATGATCCGTTGTCGGAAGAGGACGTGAAGAAGATCAAGGACAATT
ATGATAAAATCTGGGATGGTGTATGGAACTCCGCTCTGGCATTTGGTCTGAATGAGAGCACCGTCCCGGCTAT
GAGCGTTTCTGCGGAGGAACGTAAAGCGGTTTTCGAAAAAGCGTGGCAGACGGGTGGCGGGTTTCGTTTTAT
GTTCGAAACCTTTGGCGACATTGCGACGAACATGGAAGCGAACATCGAGGCGCAAAATTTTATTAAAGGTAAA
ATCGCGGAGATTGTGAAAGACCCCGCGATTGCGCAAAAATTAATGCCGCAGGATCTGTACGCTAAACGTCCGC
TGTGTGATAGCGGTTATTACAACACGTTCAACCGCGACAACGTTCGCCTGGAGGATGTTAAGGCCAACCCGAT
TGTTGAGATCACCGAAAACGGCGTGAAGTTGGAGAACGGTGACTTCGTGGAGCTGGATATGTTGATCTGCGC
CACGGGTTTTGACGCGGTTGATGGTAACTACGTTCGCATGGACATCCAAGGCAAAAACGGCCTGGCTATGAA
AGATTATTGGAAAGAAGGTCCGTCCAGCTACATGGGCGTGACCGTTAATAACTACCCGAACATGTTCATGGTTC
TGGGCCCAAACGGTCCGTTTACCAATCTGCCTCCGTCCATCGAGAGCCAGGTGGAGTGGATTAGCGATACCA
TTCAATATACTGTGGAGAACAACGTCGAGAGCATTGAGGCGACCAAAGAAGCGGAGGAGCAGTGGACCCAAA

CGTGCGCAAACATTGCTGAAATGACCCTGTTTCCGAAAGCGCAGTCCTGGATTTTCGGCGCGAACATCCCGG
GCAAAAAGAACACCGTGTACTTCTACCTGGGTGGGTTAAAGGAGTATCGTAGCGCGTTAGCGAATTGTAAGAA
TCATGCATATGAAGGCTTCGACATCCAGCTGCAGCGCTCCGATATCAAGCAGCCGGCGAACGCCTAA

Amino acid sequence (577):

MGSSHHHHHHSSGLVPRGSHMASMTGGQQMGRGSMSQKMDFDAIVIGGGFGGLYAVKKLRDELELKVQAFDKA
TDVAGTWYWNRYPGALTDTETHLYCYSWDKELLQSLEIKKKYVQGPDVRKYLQQVAEKHDLKKSYQFNTAVQSA
HYNEADALWEVTTEYGDKYTARFLITALGLLSAPNLPNIKGINQFKGELHHTSRWPDDVSFEGKRVGVIGTGSTGV
QVITAVAPLAKHLTVFQRSAQYSVPIGNDPLSEEDVKKIKDNYDKIWDGVWNSALAFGLNESTVPAMSVSAEERKA
VFEKAWQTGGGFRFMFETFGDIATNMEANIEAQNFIKGKIAEIVKDPAIAQKLMPQDLYAKRPLCDSGYYNTFNRD
NVRLEDVKANPIVEITENGVKLENGDFVELDMLICATGFDAVDGNYVRMDIQGKNGLAMKDYWKEGPSSYMGVTV
NNYPNMFMVLGPNGPFTNLPPSIESQVEWISDTIQYTVENNVESIEATKEAEEQWTQTCANIAEMTLFPKAQSWIF
GANIPGKKNTVYFYLGGLKEYRSALANCKNHAYEGFDIQLQRSDIKQPANA

Cyclohexanone monooxygenase from *Acinetobacter* sp. NCIMB 9871 (Acinetobacter johnsonii) (mutation L3-4)[1] on a pRSF_Duet with tag (C-His) (Accession number: GenBank: AB006902.2) Base sequence (codon-optimized for *E. coli*): pRSF-mAcinetoCHMO

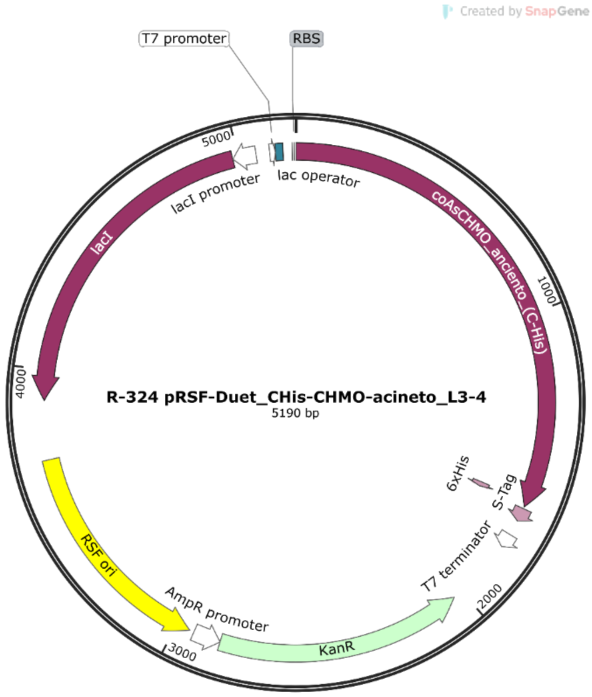

Base sequence (1650 bp):

ATGTCACAAAAAATGGATTTTGATGCTATCGTGATTGGTGCGGGTTTTGGCGGACTTTATGCAGTCAAAAAATT
AAGAGACGAGCTCGAACTTAAGGTTCAGGCTTTTGATAAAGCCACGGATGTCGCAGGTACTTGGTACTGGAAC
CGTTACCCAGGTGCATTGACGGATACAGAAACCCACCTCTACTGCTATTCTTGGGATAAAGAATTACTACAATC
GCTAGAAATCAAGAAAAAATATGTGCAAGGCCCTGATGTACGCAAGTATTTACAGCAAGTGGCTGAAAAGCATG
ATTTAAAGAAGAGCTATCAATTCAATACCGCGGTTCAATCGGCTCATTACAACGAAGCAGATGCCTTGTGGGAA
GTCACCACTGAATATGGTGATAAGTACACGGCGCGTTTCCTCATCACTGCTTTAGGCTTATTGTCTGCGCCTAA
CTTGCCAAACATCAAAGGCATTAATCAGTTTAAAGGTGAGCTGCATCATACCAGCCGCTGGCCAGATGACGTA

AGTTTTGAAGGTAAACGTGTCGGCGTGATTGGTACGGGTTCCACCGGTGTTCAGGTTATTACGGCTGTGGCA
CCTCTGGCTAAACACCTCACTGTCTTCCAGCGTTCTGCACAATACAGCGTTCCAATTGGCAATGATCCACTGT
CTGAAGAAGATGTTAAAAAGATCAAAGACAATTATGACAAAATTTGGGATGGTGTATGGAATTCAGCCCTTGCC
TTTGGCCTGAATGAAAGCACAGTGCCAGCAATGAGCGTATCAGCTGAAGAACGCAAGGCAGTTTTTGAAAAG
GCATGGCAAACAGGTGGCGGTTTCCGTTTCATGTTTGAAACTTTCGGTGATATTGCCACCAATATGGAAGCCA
ATATCGAAGCGCAAAATTTCATTAAGGGTAAAATTGCTGAAATCGTCAAAGATCCAGCCATTGCACAGAAGCTT
ATGCCACAGGATTTGTATGCAAAACGTCCGTTGTGTGACAGTGGTTACTACGAGACCTTTAACCGTGACAATG
TCCGTTTAGAAGATGTGAAAGCCAATCCGATTGTTGAAATTACCGAAAACGGTGTGAAACTCGAAAATGGCGA
TTTCGTTGAATTAGACATGCTGATATGTGCCACAGGTTTTGATGCCGTCGATGGCAACTATGTGCGCATGGACA
TTCAAGGTAAAAACGGCTTGGCCATTAAAGACTACTGGAAAGAAGGTCCGTCGAGCTATATGGGTGTCACCGT
AAATAACTATCCAAACATGTTCATGGTGCTTGGACCGAATGGCCCGTTTACCAACCTGCCGCCATCAATTGAAT
CACAGGTGGAATGGATCAGTGATACCATTAAATACGCGGAGGAAAACAATGTTGAATCCATTGAAGCGACAAA
AGAAGCGGAAGAACAATGGACTCGTACTTGCGCCGAGATTGCGGAAATGACCTTATTCCCTAAAGCGCAATCC
TGGATTTTTGGTGCGAATATCCCGGGCAAGAAAAACACGGTTTACTTCTATCTCGGTGGTTTAAAAGAATATCG
CAGTGCGCTAGCCAACTGCAAAAACCATGCCTATGAAGGTTTTGATATTCAATTACAACGTTCAGATATCAAGC
AACCTGCCAATGCCCACCACCACCACCACCACTAA

Amino acid sequence (549):

MSQKMDFDAIVIGAGFGGLYAVKKLRDELELKVQAFDKATDVAGTWYWNRYPGALTDTETHLYCYSWDKELLQSL
EIKKKYVQGPDVRKYLQQVAEKHDLKKSYQFNTAVQSAHYNEADALWEVTTEYGDKYTARFLITALGLLSAPNLPNI
KGINQFKGELHHTSRWPDDVSFEGKRVGVIGTGSTGVQVITAVAPLAKHLTVFQRSAQYSVPIGNDPLSEEDVKKI
KDNYDKIWDGVWNSALAFGLNESTVPAMSVSAEERKAVFEKAWQTGGGFRFMFETFGDIATNMEANIEAQNFIKG
KIAEIVKDPAIAQKLMPQDLYAKRPLCDSGYYETFNRDNVRLEDVKANPIVEITENGVKLENGDFVELDMLICATGFD
AVDGNYVRMDIQGKNGLAIKDYWKEGPSSYMGVTVNNYPNMFMVLGPNGPFTNLPPSIESQVEWISDTIKYAEEN
NVESIEATKEAEEQWTRTCAEIAEMTLFPKAQSWIFGANIPGKKNTVYFYLGGLKEYRSALANCKNHAYEGFDIQL
QRSDIKQPANAHHHHHH

Transformation of plasmids

For the transformation of plasmids, 30 ng of plasmid (for two plasmids 2 x 30 ng each) were first added to 50 µL of frozen, chemically competent *E. coli* BL21(DE3) cells. These were then incubated on ice for 30 minutes. This was followed by heat shock for 90 seconds at 42 °C to transfer the plasmid into the cells. This was followed by another incubation on ice for 10 minutes. LB medium (1 mL) was added to the cells and incubated for 3 hours at 37 °C. To concentrate the cells, they were first centrifuged at 7000 x g for 2 minutes and the pellet was resuspended in 100 µL of LB medium. The suspension was plated out on an LB agar plate (with appropriate antibiotic) and incubated at 37 °C overnight in an incubator.

Expression of ADH and CHMO

Expression was performed adapted from reference [22]. Expression of the enzymes was performed in *E. coli* BL21 (DE3). For the preparation of the preculture containing ADH, 5 mL of LB medium (with appropriate antibiotic) was inoculated in a culture tube with a colony of LB agar plate. Incubation took place overnight at 37 °C and 180 rpm. For the preparation of the preculture containing CHMO, 20 mL of LB medium (with appropriate antibiotic) was

inoculated in a buffled flask (100 mL volume) with a colony of LB agar plate. Incubation took place overnight at 37 °C and 120 rpm. Subsequently, expression of the main culture was performed in TB medium.

Expression in TB-medium

Inoculation of the main culture (1000 mL TB medium in a 5 L flask with appropriate antibiotic) was performed with the preculture (1%). The main culture was subsequently incubated at 37 °C and 180 rpm. At an OD600 between 0.4 and 0.6, isopropyl-β-D-thiogalactoside (IPTG, 0.5 mM) was added for the expression of the recombinant protein. The main culture was incubated at 25 °C and 180 rpm for 20 h. Cells were harvested by centrifugation at 4000 x g and 4 °C for 30 min. Cell pellets were stored at -20 °C or further processed into crude extract.

A suspension of cells and potassium phosphate buffer (PPB, 50 mM) at pH 7 (1/3 *v*/*v*) was prepared and sonicated (Bandelin Sonopuls HD 2070) with 3× 3 min bursts (five cycles, 20% energy) and a 3 min incubation on ice in between. The suspension was then centrifuged at 21000 x g for 30 min at 4°C to obtain the crude extract as supernatant. Protein concentration was determined by the Bradford assay.

Cells containing CHMO: Inoculation of the main culture (200 mL TB medium in a 1L buffled flask with appropriate antibiotic) was performed with the preculture (1%). The main culture was subsequently incubated at 37 °C and 120 rpm. At an OD600 between 0.4 and 0.6, isopropyl-β-D-thiogalactoside (IPTG, 0.05 mM) was added for the expression of the recombinant protein. The main culture was incubated at 25 °C and 120 rpm for 20 h. Cells were harvested by centrifugation at 4000 x g and 4 °C for 30 min. The cell pellets were stored at -20 °C.

Cell pellets were lyophilized for at least 1 night (Lyovapor™ L-200, Büchi). On average, whole cells lose 3/4 of their weight during the drying process.

4. Synthesis and Biotransformation

Hydrogenation of biobased phenol

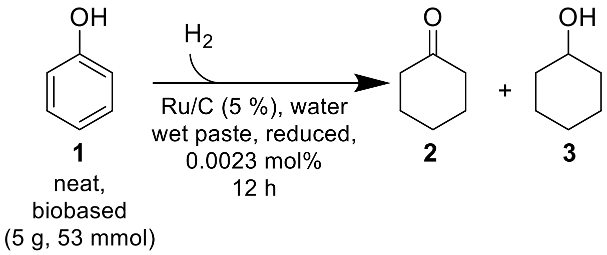

The hydrogenation reactions were performed in a Parr Series 5000 Multiple Reactor System. In the multiple reactor system, up to six reactions could be carried out at the same time, each with a maximum volume of 75 mL and a stirring rate of 1000 rpm. For the hydrogenation of phenol **1**, biobased phenol **1** (4.67 mL, 5 g, neat) was placed in a reactor. This was followed by the addition of ruthenium, 5% on activated carbon, reduced, 50% water wet paste (5 mg, 0.0023 mol%). The phenol was then melted in an autoclave. After evacuation and flushing of the apparatus with nitrogen (3 x) and hydrogen (3 x), hydrogen (defined pressure) was added to the apparatus and the suspension was then stirred for 12 h at a defined temperature. After 12 h, the reaction mixture was cooled to room temperature and then the suspension was filtered over a syringe filter (0.2 µm) and the product was analyzed via ^{1}H-NMR and GC. The reactions were carried out in duplicate.

Phenol **1**: ^{1}H NMR (500 MHz, Chloroform-*d*) δ 7.30 – 7.22 (m, 2H), 6.99 – 6.91 (m, 1H), 6.88 – 6.81 (m, 2H), 4.87 (s, 1H).

Cyclohexanone **2**: ^{1}H NMR (500 MHz, Chloroform-*d*) δ 2.24 (t, *J* = 6.7 Hz, 6H), 1.77 (d, *J* = 17.5 Hz, 1H), 1.67 – 1.59 (m, 3H).

Cyclohexanol **3**: ^{1}H NMR (500 MHz, Chloroform-*d*) δ 3.55 (dp, *J* = 8.7, 4.1 Hz, 1H), 2.15 (s, 1H), 1.85 (dt, *J* = 10.9, 5.2 Hz, 2H), 1.71 (dd, *J* = 7.8, 4.0 Hz, 2H), 1.50 (dt, *J* = 12.9, 3.8 Hz, 1H), 1.23 (td, *J* = 10.8, 4.7 Hz, 4H), 1.14 (ddd, *J* = 15.4, 12.9, 7.3 Hz, 1H).

The data corresponds to the ones reported in literature.

Table S2: Conditions and results of the conversions of the hydrogenation of biobased phenol. Results shown as a percentage in the crude reaction mixture, analyzed via GC.

entry	T / °C	p / bar	**1** / %	**2** / %	**3** / %
1	60	120	5 ± 0	0 ± 0	95 ± 0
2	60	130	5 ± 1	1 ± 0	94 ± 1
3	70	120	1 ± 1	0 ± 1	99 ± 1
4	70	130	0 ± 0	0 ± 0	>99 ± 0
5	80	100	0 ± 1	0 ± 0	>99 ± 1

Activity assay of ADHs

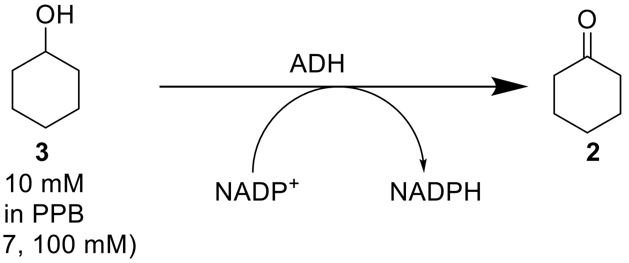

3
10 mM
in PPB
(pH 7, 100 mM)

2

t = 0 h or t = 2 h

The activity assay was performed adapted from reference [22]. To determine the activity [U/mL], specific activity [U/mg] and deactivation of the different ADHs in buffer, the consumption of the cofactor $NADP^+$ during the biotransformation of cyclohexanol (**3**) to cyclohexanone (**2**) was studied at 30°C and 340 nm for 60 seconds using the Tecan Reader Spark 10M. The activity was determined by measuring the absorption at 340 nm (absorption maximum of NADPH). $NADP^+$ consumption leads to the formation of NADPH, thus the absorption increases linearly.

In detail, cyclohexanol (**3**, 10 mM) was oxidized using the crude extract of ADH (25 µL of a 1:100 dilution) and $NADP^+$ (0.22 mM) in PPB (100 mM, pH 7). The total volume is 250 µL. The assay was performed in quadruplicate.

Using this linear fit, the activity can be determined using the following equation:

$$A = (\Delta E \cdot V_t \cdot f)/(\varepsilon \cdot V_E \cdot d)$$

The enzyme activity is given in A (U mL^{-1}), the time depending change in extinction in ΔE (1 min^{-1}), the total volume in V_t (250 µL), the volume of enzyme solution in V_E (25 µL), the dilution factor f (100), the extinction coefficient ε of NADPH at 340 nm (6.300 L mol^{-1} cm^{-1}) and the layer thickness d (8 mm).

The specific activity is determined by dividing the activity by the protein concentration, as determined through the Bradford assay.

In addition, activity assays were performed after incubation of ADH in PPB (100 mM, pH 7) at 25°C for 2 hours.

Table S3: Activities and specific activities of the different ADHs determined by spectrophotometric assay.

ADH	Protein concentration [mg mL^{-1}]	acitivity 0 h [U mL^{-1}]	acitivity 2 h [U mL^{-1}]	Spec. acitivity 0 h [U mg^{-1}]	Spec. acitivity 2 h [U mg^{-1}]
Lk	21.17	5.38	2.42	0.26	0.12
Tb	10.26	1.78	1.92	0.16	0.17
Te	18.03	0.67	0.73	0.04	0.04

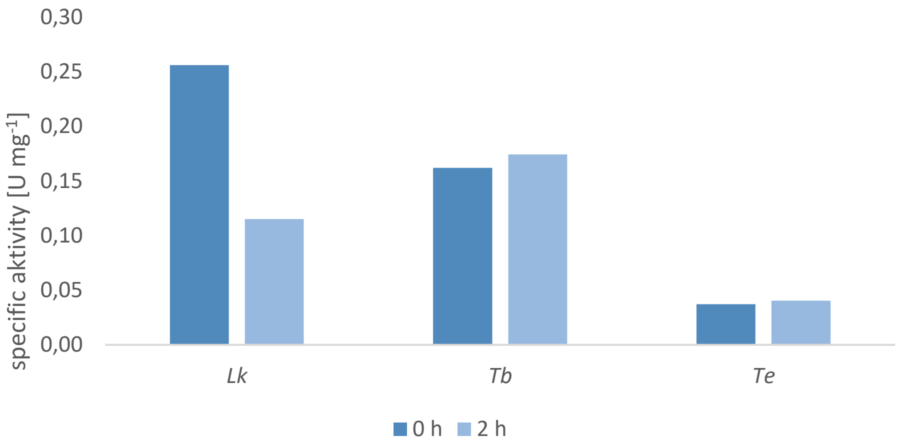

Figure S2: Initial specific activities and specific activities of the different ADHs after 2 h incubation determined by spectrophotometric assay.

Analytical biotransformations to determine optimal vector system in aqueous media

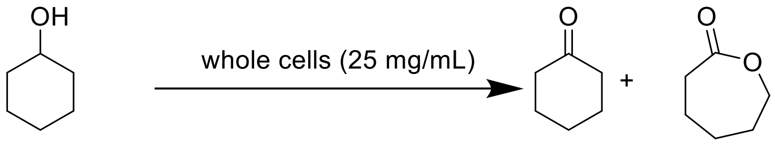

3 40 mM in PPB (50 mM, pH 7) **2** **4**

For the double oxidation of cyclohexanol to ε-caprolactone, cyclohexanol (40 mM) and bio wet mass (25 mg/mL, *E. coli* BL21(DE3)- A: pRSF-AcinetoCHMO & pACYC-LkADH, B: pRSF-mAcinetoCHMO & pACYC-LkADH, C: pRSF-AcinetoCHMO & pET21a-LkADH) were added to PPB (0.5 mL, pH 7, 50 mM) in 2 mL Eppendorf tubes (sealed with aeroseal foil). The reactions were incubated for 24 hours at 25°C and 850 rpm. Ethyl acetate (3 x 500 µL) was then added, the Eppendorf tubes were centrifuged, and the organic phase was collected (3 x).

The organic phase was analyzed by GC. Each reaction was carried out in duplicate.

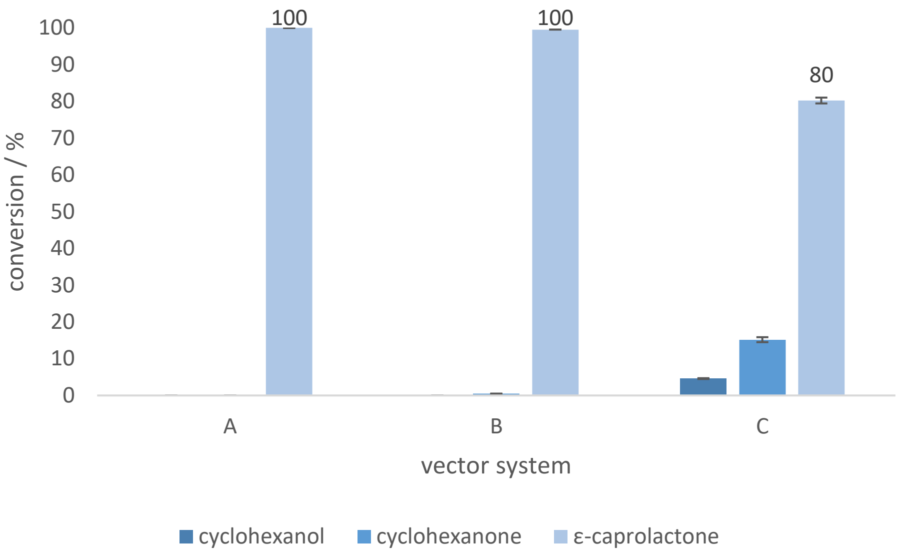

Figure S3: Results of the enzymatic double oxidation using different vector system combinations. Results shown as a percentage in the crude reaction mixture, analyzed via GC.

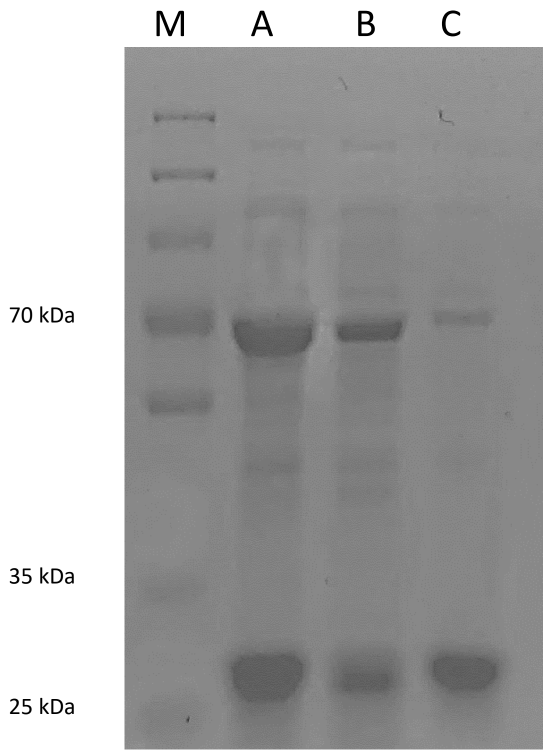

Figure S4: SDS-PAGE of the crude extracts of different vector system combinations: *E. coli* BL21(DE3)- A: pRSF-AcinetoCHMO & pACYC-LkADH, B: pRSF-mAcinetoCHMO & pACYC-LkADH C: pRSF-AcinetoCHMO & pET21a-LkADH.

Initial biotransformation in cyclohexane

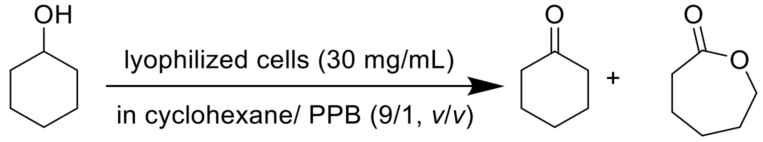

3 **2** **4**
40 mM

For double oxidation of cyclohexanol to ε-caprolactone, lyophilized cells (*E. coli* BL21(DE3)- pRSF-AcinetoCHMO (A) or pRSF-mAcinetoCHMO (B) & pACYC-LkADH, 30 mg/mL, lyophilized) were placed in cyclohexane (450 µL, containing 40 mM cyclohexanol) in 2 mL Eppendorf tubes, PPB (100 mM, pH 7, 50 µL, containing 40 mM cyclohexanol) was added and covered with oxygen. The reactions were incubated for 17 h at 25°C and 850 rpm. Ethyl acetate (0.5 mL) was then added, the Eppendorf tubes were centrifuged (20000 x g, 2 min), and the organic phase was collected and analyzed via GC. Each reaction was carried out in duplicate.

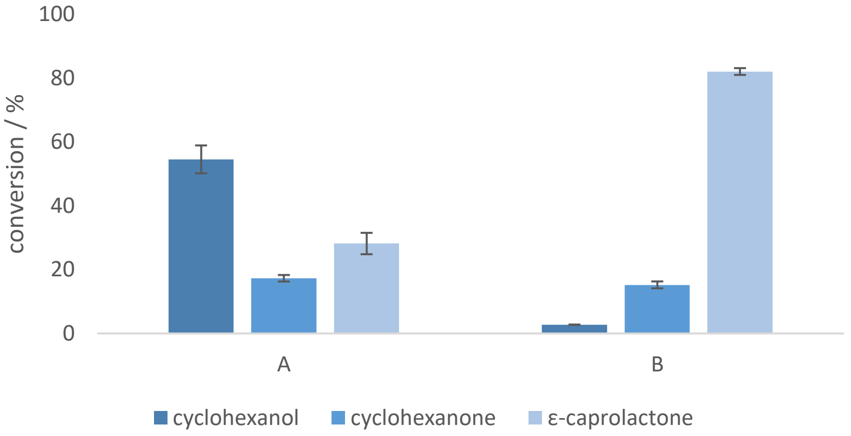

Figure S5: Results of the initial enzymatic double oxidation in organic media using different vector system combinations. Results shown as a percentage in the crude reaction mixture, analyzed via GC.

Screening of different solvents for double-oxidation of cyclohexanol

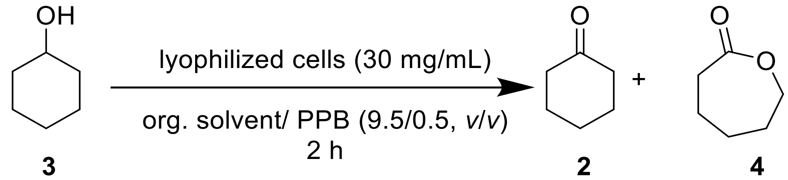

3 **2** **4**
40 mM

For the double oxidation of cyclohexanol to ε-caprolactone, lyophilized cells (*E. coli* BL21(DE3)- pRSF-mAcinetoCHMO & pACYC-LkADH, 30 mg/mL) were placed in different solvents (450 µL, containing 40 mM cyclohexanol) in 2 mL Eppendorf tubes, PPB (100 mM, pH 7, 50 µL, containing 40 mM cyclohexanol) was added and covered with oxygen. Reactions were incubated for 2 hours at 25°C and 850 rpm. Ethyl acetate (500 µL) was then added, the Eppendorf tubes were centrifuged (20000 x g, 2 min), and the organic phase was collected and analyzed by GC. Each reaction was run in duplicate.

Table S4: Results of the enzymatic double oxidation in organic media for screening the optimal solvent. Results shown as a percentage in the crude reaction mixture, analyzed via GC.

entry	solvent	cyclohexanol / %	cyclohexanone / %	ε-caprolactone / %
1	cyclohexane	32 ± 2	12 ± 0	56 ± 2
2	diethyl carbonate	88 ± 0	8 ± 0	4 ± 0
3	methyl *tert*-butyl ether	37 ± 1	6 ± 0	57 ± 1
4	2,4-dimethyl-3-pentanone	42 ±1	48 ± 1	10 ± 0
5	ethyl acetate	90 ±1	7 ± 1	3 ± 0
6	isopropyl acetate	25 ± 0	4 ± 0	71 ± 0
7	*n*-butyl acetate	10 ± 0	3 ± 0	86 ± 0
8	ethyl butyrate	32 ± 2	4 ± 0	64 ± 2
9	ethyl propionate	100 ± 0	0 ± 0	0 ± 0
10	mixture of esters[a]	91 ± 0	4 ± 0	5 ± 0
11	4-methyl-2-pentanone[b]	36 ± 2	62 ± 2	2 ± 0

[a] Mixture of ethyl acetate, ethyl propionate and ethyl butyrate (1/1/1, v/v/v). [b] Oxidation of the solvent 4-methyl-2-pentanone to the corresponding ester cannot be excluded based on this initial experiment.

Negative controls with empty vectors in organic media

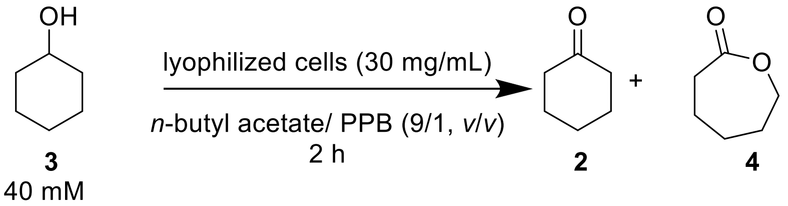

For the double oxidation of cyclohexanol, lyophilized cells (designated as A: *E. coli* BL21(DE3)- pRSF-mAcinetoCHMO & pACYC-LkADH, or B: *E. coli* BL21(DE3)- pRSF-empty & pACYC-empty 30 mg/mL) were placed in *n*-butyl acetate (450 µL, containing 40 mM cyclohexanol) in Eppendorf tubes. Then, PPB (100 mM, pH 7, 50 µL, containing 40 mM cyclohexanol) was added, and oxygen was supercharged. The reactions were incubated for two hours at a temperature of 25°C and a speed of 850 rpm. Ethyl acetate (500 µL) was added, followed by centrifugation of the Eppendorf tubes at 20000 x g for 2 min. The resulting organic phase was collected. GC analysis was performed. All reactions were performed in duplicate.

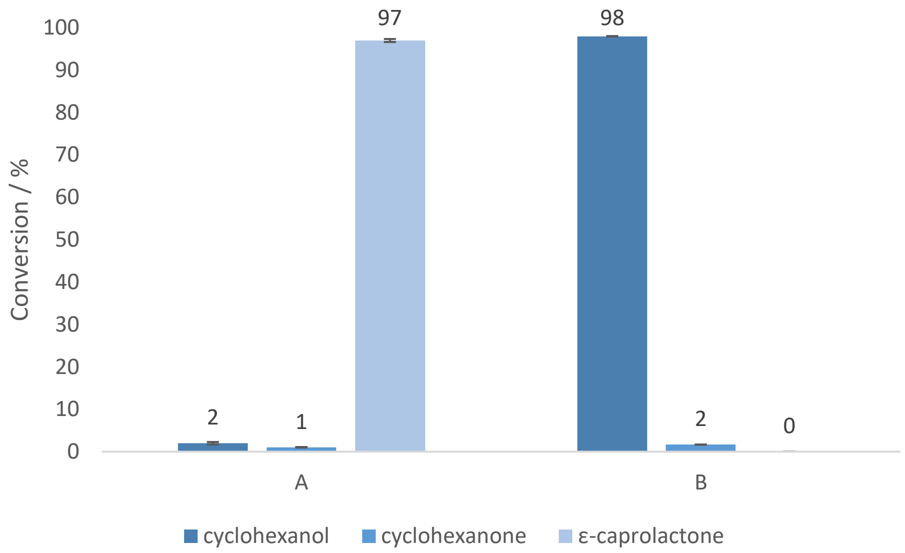

Figure S6: Results of the enzymatic double oxidation in organic media with lyophilized cells vs. lyophilized cells containing empty vector systems. Results shown as a percentage in the crude reaction mixture, analyzed via GC.

Suitability of lyophilized cells vs. wet biomass

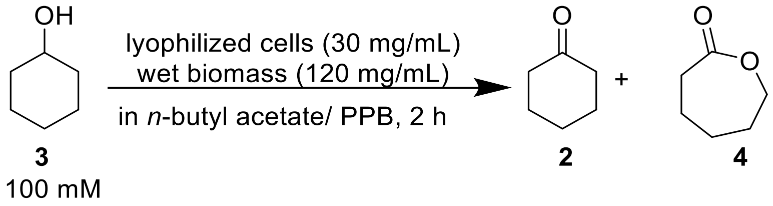

For the double oxidation of cyclohexanol to ε-caprolactone, A: lyophilized cells (*E. coli* BL21(DE3)- pRSF-mAcinetoCHMO & pACYC-LkADH, 30 mg/mL) or B: wet biomass (*E. coli* BL21(DE3)- pRSF-mAcinetoCHMO & pACYC-LkADH, 120 mg/mL) in *n*-butyl acetate (A, B: 450 µL, containing A: 100 mM cyclohexanol or B: 110 mM cyclohexanol) in 2 mL Eppendorf tubes, PPB (100 mM, pH 7, A: 50 µL, B: 5 µL, containing A: 100 mM cyclohexanol or B: 110 mM cyclohexanol) was added and covered with oxygen. The biotransformations were incubated for 2 hours at 25°C and 850 rpm. Ethyl acetate (1250 µL) was then added, the Eppendorf tubes were centrifuged (20000 x g, 2 min), and the organic phase was collected and analyzed by GC. Each reaction was run in duplicate.

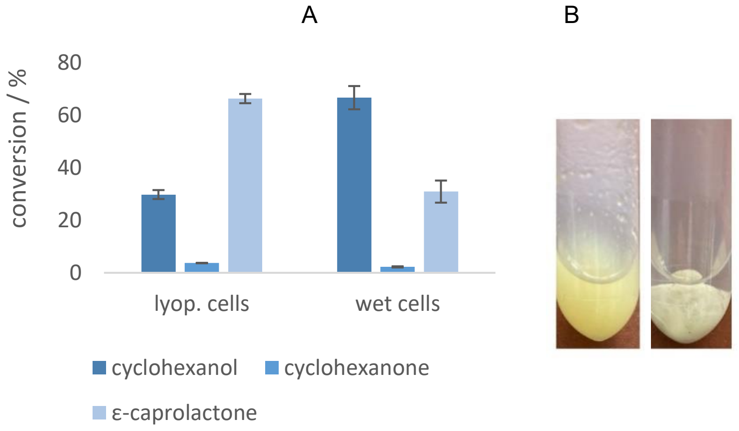

Figure S7: A: Results of the enzymatic double oxidation in organic media using lyophilized cells vs. wet biomass. Results shown as a percentage in the crude reaction mixture, analyzed via GC. B: Photos of the analytic biotransformations; left: Reaction using lyophilized cells; right: Reaction using wet biomass.

Double oxidation of cyclohexanol (40 respectively 80 mM) in *n*-butyl acetate

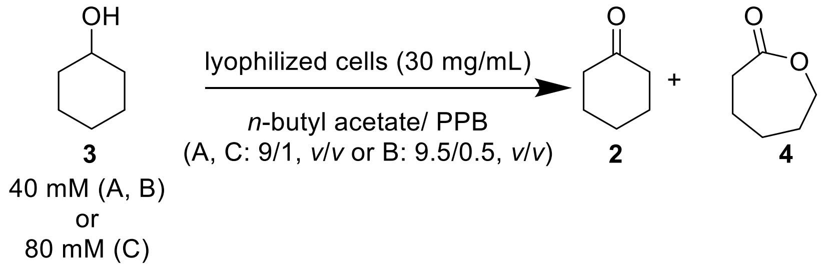

The lyophilized cells (*E. coli* BL21(DE3)- pRSF-mAcinetoCHMO & pACYC-LkADH, 30 mg/mL) were utilized for the double oxidation of cyclohexanol to ε-caprolactone. The cells were placed in Eppendorf tubes containing *n*-butyl acetate (A, C: 450 or B: 475 µL, containing 40 mM (A, B) or 80 mM (C) cyclohexanol). Subsequently, PPB (100 mM, pH 7, A, C: 50 or B: 25 µL, containing 40 mM (A, B) or 80 mM (C) cyclohexanol) was added, and the reaction was covered with oxygen. The incubation process continued for different times at 25°C and 850 rpm. Ethyl acetate (500 µL (A, B) or 1500 µL (C)) was added, followed by centrifugation of the Eppendorf tubes at 20000 x g for 2 min. The resulting organic phase was collected. GC analysis was performed. All reactions were performed in duplicate.

Table S5: A: Results of the time dependent conversion of cyclohexanol (40 mM) in *n*-butyl acetate (10 % buffer addition). Results shown as a percentage in the crude reaction mixture, analyzed via GC.

**A**	time / h	cyclohexanol / %	cyclohexanone / %	ε-caprolactone / %
	2 h	2 ± 0	1 ± 0	97 ± 0
	4 h	0 ± 0	3 ± 0	97 ± 0

Table S6: Results of the time dependent conversion of cyclohexanol (40 mM) in *n*-butyl acetate (5 % buffer addition). Results shown as a percentage in the crude reaction mixture, analyzed via GC.

**B**	time / h	cyclohexanol / %	cyclohexanone / %	ε-caprolactone / %
	2 h	13 ± 0	4 ± 0	83 ± 1
	4 h	6 ± 1	4 ± 0	91 ± 1
	6 h	4 ± 0	3 ± 0	93 ± 0

Table S7: Results of the time dependent conversion of cyclohexanol (80 mM) in *n*-butyl acetate (10 % buffer addition). Results shown as a percentage in the crude reaction mixture, analyzed via GC.

**C**	time / h	cyclohexanol / %	cyclohexanone / %	ε-caprolactone / %
	2 h	22 ± 0	4 ± 0	74 ± 0
	4 h	10 ± 1	4 ± 0	86 ± 1
	6 h	5 ± 1	3 ± 0	92 ± 1

Double oxidation of cyclohexanol (100 respectively 200 mM) in *n*-butyl acetate

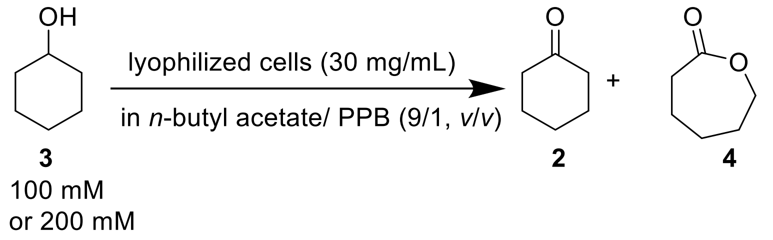

The lyophilized cells (*E. coli* BL21(DE3)- pRSF-mAcinetoCHMO & pACYC-LkADH, 30 mg/mL) were utilized for the double oxidation of cyclohexanol to ε-caprolactone. The cells were placed in Eppendorf tubes containing *n*-butyl acetate (450 µL, containing 100 mM or 200 mM cyclohexanol). Subsequently, PPB (100 mM, pH 7, 50 µL, containing 100 mM or 200 mM cyclohexanol) was added, and the reaction was covered with oxygen. The incubation process continued for varying durations at 25°C and 850 rpm. Ethyl acetate (1250 µL (100 mM) or 1500 µL (200 mM)) was added, followed by centrifugation of the Eppendorf tubes at 20000 x g for 2 min. The resulting organic phase was collected. GC analysis was performed. All reactions were performed in duplicate.

Table S8: Results of the time dependent conversion of cyclohexanol (100 mM) in *n*-butyl acetate (10 % buffer addition). Results shown as a percentage in the crude reaction mixture, analyzed via GC.

100 mM	time / h	cyclohexanol / %	cyclohexanone / %	ε-caprolactone / %
	2	27 ± 1	4 ± 0	69 ± 1
	4	15 ± 1	5 ± 0	80 ± 1
	6	11 ± 2	5 ± 0	84 ± 2
	8	10 ± 2	5 ± 0	85 ± 3
	12	8 ± 0	5 ± 0	87 ± 0
	16	9 ± 0	5 ± 0	86 ± 1
	24	6 ± 1	5 ± 0	89 ± 1

Table S9: Results of the time dependent conversion of cyclohexanol (100 mM) in *n*-butyl acetate (10 % buffer addition). Results shown as a molarities in the crude reaction mixture, analyzed via GC.

100 mM	time / h	cyclohexanol / mM	cyclohexanone / mM	ε-caprolactone / mM
	2	27 ± 1	4 ± 0	70 ± 1
	4	15 ± 0	5 ± 0	81 ± 2
	6	11 ± 2	5 ± 0	85 ± 2
	8	10 ± 2	5 ± 0	88 ± 2
	12	7 ± 0	5 ± 0	86 ± 0
	16	9 ± 0	5 ± 0	86 ± 0
	24	6 ± 1	5 ± 0	89 ± 0

Table S10: Results of the time dependent conversion of cyclohexanol (200 mM) in *n*-butyl acetate (10 % buffer addition). Results shown as a percentage in the crude reaction mixture, analyzed via GC.

200 mM	time / h	cyclohexanol / %	cyclohexanone / %	ε-caprolactone / %
	2	63 ± 1	4 ± 0	33 ± 1
	4	60 ± 5	4 ± 1	36 ± 4
	6	50 ± 1	6 ± 0	44 ± 1
	8	49 ± 0	6 ± 0	46 ± 0
	16	45 ± 1	8 ± 0	47 ± 2
	24	46 ± 2	7 ± 0	46 ± 2

Table S11: Results of the time dependent conversion of cyclohexanol (200 mM) in *n*-butyl acetate (10 % buffer addition). Results shown as a molarities in the crude reaction mixture, analyzed via GC.

200 mM	time / h	cyclohexanol / mM	cyclohexanone / mM	ε-caprolactone / mM
	2	128 ± 0	8 ± 0	66 ± 3
	4	122 ± 15	9 ± 2	73 ± 5
	6	102 ± 3	12 ± 0	89 ± 2
	8	101 ± 1	12 ± 0	95 ± 0
	16	93 ± 1	16 ± 0	98 ± 5
	24	92 ± 2	15 ± 0	93 ± 5

Biotransformation in *n*-butyl acetate - Initial reaction rates

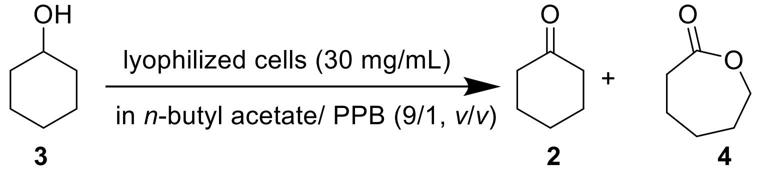

A: 100 mM
B: 200 mM
C: 100 mM in presence of ε-Caprolactone (100 mM)

The lyophilized cells (*E. coli* BL21(DE3)- pRSF-mAcinetoCHMO & pACYC-LkADH, 30 mg/mL) were utilized for the double oxidation of cyclohexanol to ε-caprolactone. The cells were placed in Eppendorf tubes containing *n*-butyl acetate (450 µL, containing A: 100 mM, B: 200 mM cyclohexanol or C: 100 mM cyclohexanol and 100 mM ε-caprolactone). Subsequently, PPB (100 mM, pH 7, 50 µL, containing A: 100 mM, B: 200 mM cyclohexanol or C: 100 mM cyclohexanol and 100 mM ε-caprolactone) was added, and the reaction was covered with oxygen. The

incubation process continued for varying durations at 25°C and 850 rpm. Ethyl acetate (1250 µL) was added, followed by centrifugation of the Eppendorf tubes at 20000 x g for 2 min. The resulting organic phase was collected. GC analysis was performed. All reactions were performed in duplicate.

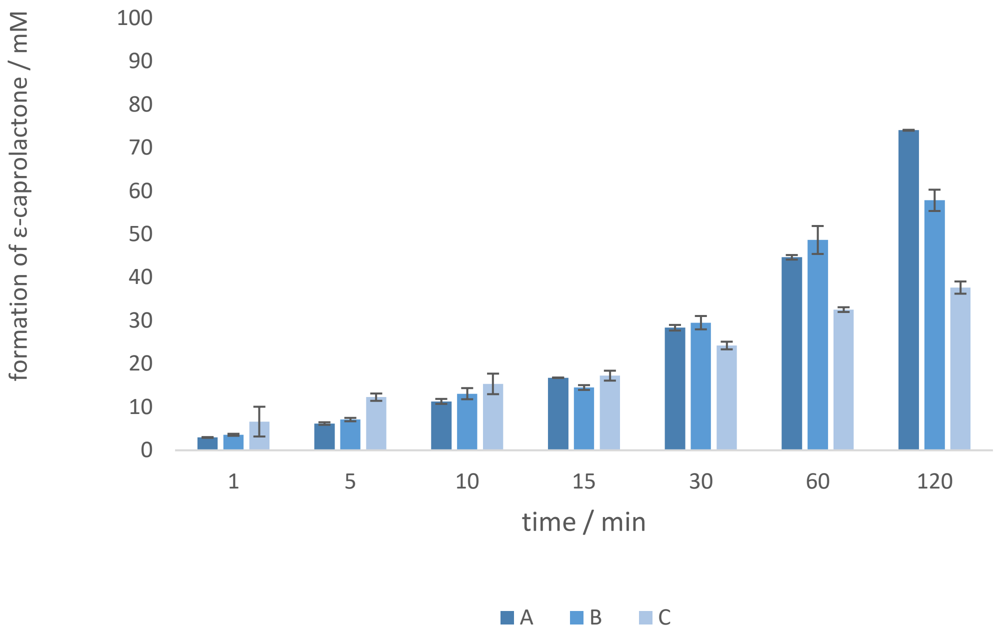

Figure S8: Results of the time dependent conversion of cyclohexanol (100 mM (A, C), 200 mM (B), C: in the presence of ε-caprolactone (100 mM)) in *n*-butyl acetate (10 % buffer addition). Results shown as a molarities in the crude reaction mixture, analyzed via GC.

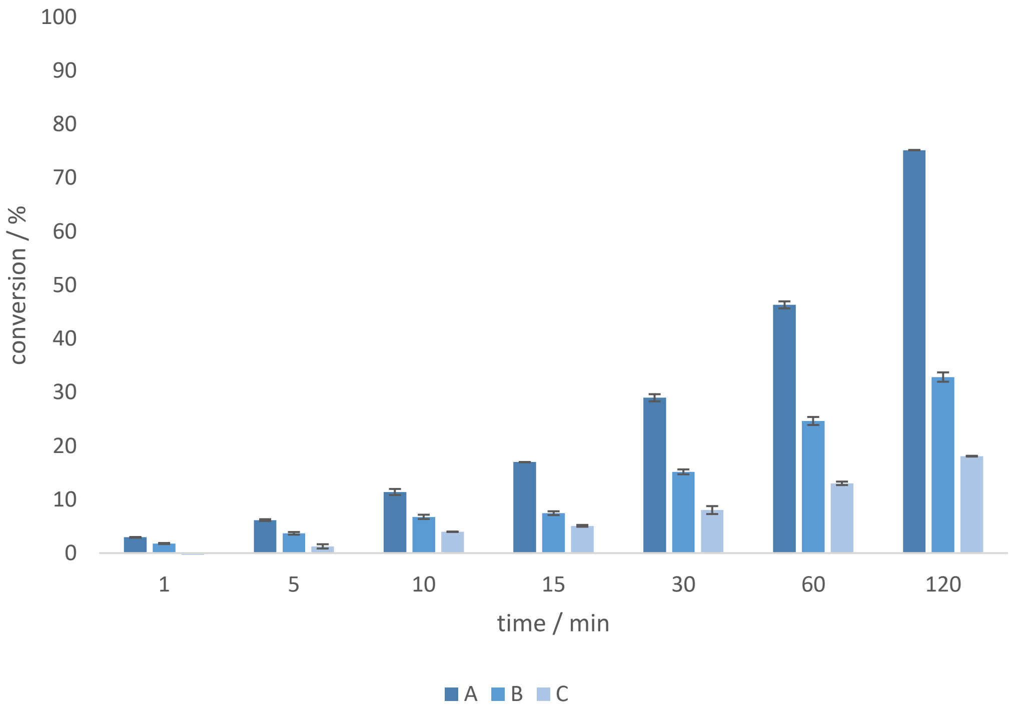

Figure S9: Results of the time dependent conversion of cyclohexanol (100 mM (A, C), 200 mM (B), C: in the presence of ε-caprolactone (100 mM)) in *n*-butyl acetate (10 % buffer addition). Results shown as a percentage in the crude reaction mixture, analyzed via GC.

Evaluation of the role of NADPH oxidase in lyophilized whole cell catalysts via spectrophotometric activity assays

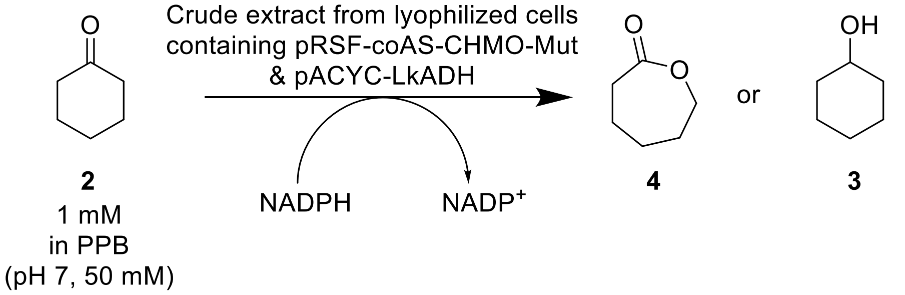

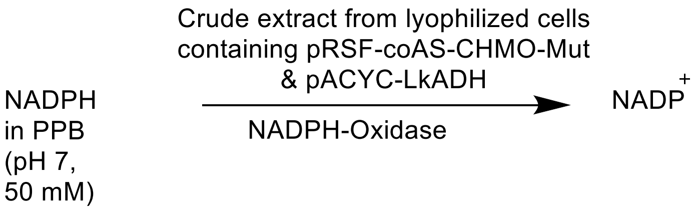

To study the role of NADPH oxidase in lyophilized whole cell catalysts containing pRSF-mAcinetoCHMO & pACYC-LkADH, we investigated the consumption of cofactor NADPH at 25°C and 340 nm for 60 seconds on Tecan Reader Spark 10M during the biotransformation of cyclohexanone to caprolactone or cyclohexanol, respectively. The activity was determined by measuring the absorption at 340 nm (absorption maximum of NADPH), allowing for the analysis of NADPH consumption through the time-dependent absorbance at 340 nm. Using the linear fit, the activity can be calculated in the assay. To perform the assay, cyclohexanone (1 mM) was reacted with the diluted crude extract of lyophilized cells (10 µL), NADPH (0.24 mM) in PPB (50 mM, pH 7) at a total volume of 250 µL. A quadruplicate determination was conducted, and negative controls were implemented without the NADPH solution or crude extract.

Moreover, activity assays were conducted without cyclohexanone to assess the NADPH oxidase activity.

Using this linear fit, the activity can be determined using the following equation:

$A = (\Delta E \cdot V_t \cdot f)/(\varepsilon \cdot V_E \cdot d)$

The enzyme activity is given in (U mL^{-1}), the time depending change in extinction in ΔE (1 min^{-1}), the total volume in V_t (250 µL), the volume of enzyme solution in V_E (10 µL), the dilution factor f, the extinction coefficient ε of NADPH at 340 nm (6.300 L mol^{-1} cm^{-1}) and the layer thickness d (8 mm).

The lyophilized cells exhibited a level of activity of 79.7 ± 6.6 U mL^{-1} in the conversion of cyclohexanone. In contrast, NADPH was converted at a rate of only 0.15 ± 0.03 U mL^{-1} when no cyclohexanone was present in the surrounding environment. Notably, all negative controls demonstrated no activity.

Stability of the lyophilized cells in *n*-butyl acetate

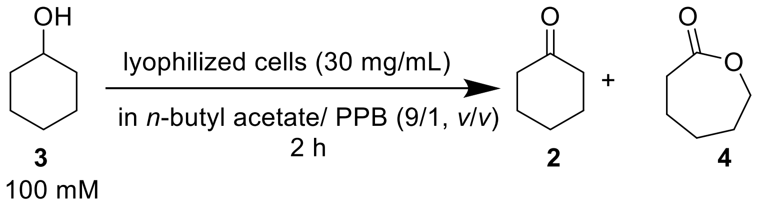

For the double oxidation of cyclohexanol (100 mM) to ε-caprolactone, lyophilized cells (*E. coli* BL21(DE3)- pRSF-mAcinetoCHMO & pACYC-LkADH, 30 mg/mL) were placed in *n*-butyl acetate (900 µL) in Eppendorf tubes. PPB (100 mM, pH 7, 100 µL) was subsequently added, and covered with oxygen. After 0, 2, and 4 hours, respectively, cyclohexanol (100 mM, 10.5 µL) was added and the biotransformations were then incubated for 2 hours at 25°C and 850 rpm. Ethyl acetate (1000 µL) was added, followed by centrifugation of the Eppendorf tubes (20000 x g, 2 min), and collection of the organic phase for GC analysis. Each reaction was performed twice.

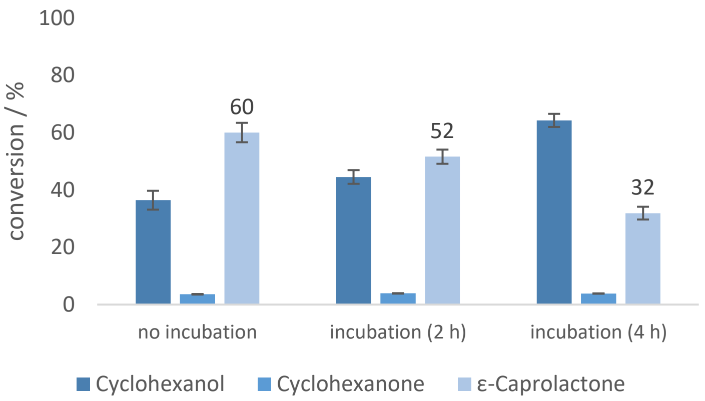

Figure S10: Results of the enzymatic double oxidation in organic media to evaluate the stability of the lyophilized cells in organic media. Results shown as a percentage in the crude reaction mixture, analyzed via GC.

Storage stability of lyophilized cells at room temperature

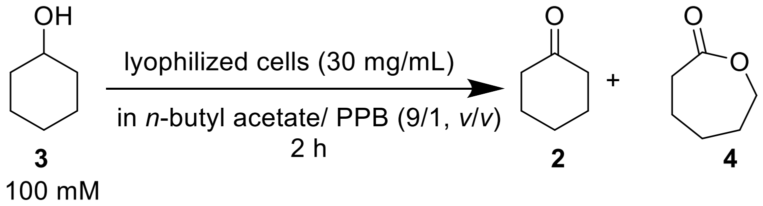

Lyophilized cells (30 mg/mL) of *E. coli* BL21(DE3)- pRSF-mAcinetoCHMO & pACYC-LkADH, taken from different batches (A-D) either before or after storage at room temperature for 5 days, were placed in Eppendorf tubes containing *n*-butyl acetate (450 µL, containing 100 mM cyclohexanol) for the double oxidation of cyclohexanol to ε-caprolactone. PPB (100 mM, pH 7, 50 µL, containing 100 mM cyclohexanol) was added and oxygen was supercoated. The reactions were incubated at 25°C and 850 rpm for 2 hours. Ethyl acetate (1250 µL) was added, followed by centrifugation of Eppendorf tubes (20000 x g, 2 min). All reactions were replicated. The organic phase was collected and subjected to analysis by GC.

Table S12: Results of the enzymatic double oxidation in organic media to evaluate the stability of the lyophilized cells at room temperature. Results shown as a percentage in the crude reaction mixture, analyzed via GC.

	cyclohexanol / %	cyclohexanone / %	ε-caprolactone / %
A	33 ± 1	4 ± 0	63 ± 1
A, stored 5 days at RT	37 ± 1	4 ± 0	59 ± 1
B	32 ± 2	3 ± 0	65 ± 2
B, stored 5 days at RT	34 ± 0	3 ± 0	63 ± 0
C	30 ± 2	4 ± 0	66 ± 2
C, stored 5 days at RT	33 ± 1	4 ± 0	63 ± 1
D	26 ± 1	4 ± 0	70 ± 1
D, stored 5 days at RT	32 ± 1	4 ± 0	64 ± 0

Biotransformation in organic media - 100 mM conversion on a 5 mL scale - glass vial vs. Falcon tube

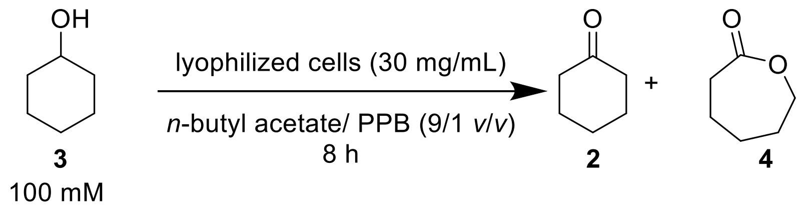

total volume 5 mL

To achieve the double oxidation of cyclohexanol to ε-caprolactone, lyophilized cells (*E. coli* BL21(DE3)- pRSF-mAcinetoCHMO & pACYC-LkADH, 30 mg/mL, 150 mg) were introduced to *n*-butyl acetate (4.5 mL, containing 100 mM cyclohexanol) in a glass vial or Falcon tube, PPB (100 mM, pH 7, 500 µL, containing 100 mM cyclohexanol) was added, and covered with oxygen. The reactions incubated for 8 hours at 25°C with 850 rpm stirring in a glass vial or 400 rpm shaking in a Falcon tube. The suspension was transferred to a Falcon tube and 5 mL of ethyl acetate was added. The tube was vortexed, centrifuged at 20000 x g for 2 minutes, and then the organic phase was isolated and analyzed by gas chromatography.

Table S13: Results of the enzymatic double oxidation in organic media in 5 mL scale to determine the impact of the reactor. Results shown as a percentage in the crude reaction mixture, analyzed via GC.

	cyclohexanol / %	cyclohexanone / %	ε-caprolactone / %
falcon tube	8	5	87
glass vial	14	4	82

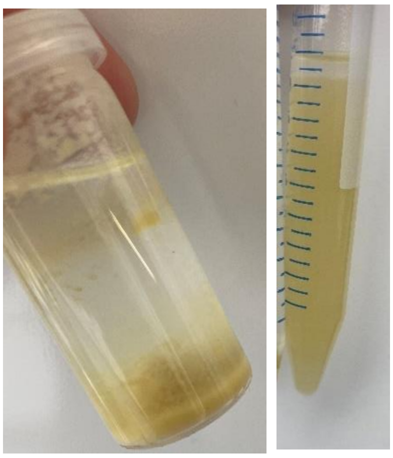

Figure S11: Photos of the enzymatic double oxidation in organic media in 5 mL scale in a glass vial (left) in contrast to the falcon tube (right) to determine the impact of the reactor.

Biotransformation in organic media - 100 mM conversion on a 10 mL scale

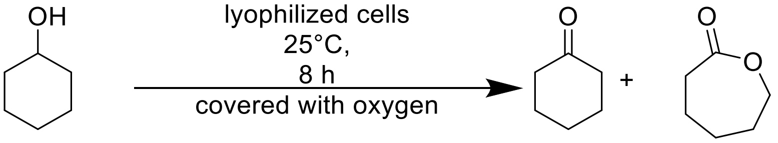

3 100 mM in *n*-butyl acetate (9 mL) and PPB (1 mL) 50 mL Falcon tube

2

4 conversion to **4**: 97 %

To achieve the double oxidation of cyclohexanol to ε-caprolactone, lyophilized cells (*E. coli* BL21(DE3)- pRSF-mAcinetoCHMO & pACYC-LkADH, 30 mg/mL, 300 mg) were introduced to *n*-butyl acetate (9 mL, containing 100 mM cyclohexanol) in a Falcon tube, PPB (100 mM, pH 7, 1 mL, containing 100 mM cyclohexanol) was added, and covered with oxygen. The reactions incubated for 8 hours at 25°C with 400 rpm shaking in a Falcon tube. Then 10 mL of ethyl acetate was added. The tube was vortexed, centrifuged at 20000 x g for 2 minutes, and then the organic phase was isolated and analyzed by gas chromatography.

Table S14: Results of the enzymatic double oxidation in organic media in 10 mL scale. Results shown as a percentage in the crude reaction mixture, analyzed via GC.

	cyclohexanol / %	cyclohexanone / %	ε-caprolactone / %
	2 ± 0	1 ± 0	97 ± 0

After removal of the solvent *in vacuo*, ε-caprolactone was isolated with an average yield of 85% (89.5 respectively 105.5 mg, purity ~ 95 %).

^{1}H NMR (500 MHz, Chloroform-*d*) δ 4.28 – 4.18 (m, 2H), 2.68 – 2.59 (m, 2H), 1.91 – 1.69 (m, 6H).

The data corresponds to the ones reported in literature.

Biotransformation in organic media - 100 mM conversion of bio-based cyclohexanol on a 10 mL scale

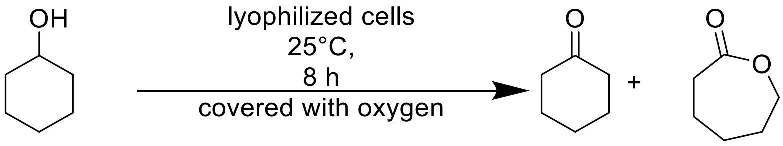

3 bio-based 100 mM in *n*-butyl acetate (9 mL) and PPB (1 mL) 50 mL Falcon tube

2

4 quantitative conversion

To achieve the double oxidation of cyclohexanol to ε-caprolactone, lyophilized cells (*E. coli* BL21(DE3)- pRSF-mAcinetoCHMO & pACYC-LkADH, 30 mg/mL, 300 mg) were introduced to *n*-butyl acetate (9 mL, containing 100 mM bio-based cyclohexanol (table S2, entry 5)) in a Falcon tube, PPB (100 mM, pH 7, 1 mL, containing 100 mM bio-based cyclohexanol (table S2, entry 5)) was added, and covered with oxygen. The reactions incubated for 8 hours at 25°C with 400 rpm shaking in a Falcon tube. Then 10 mL of ethyl acetate was added. The tube was vortexed, centrifuged at 20000 x g for 2 minutes, and then the organic phase was isolated and analyzed by gas chromatography.

Table S15: Results of the enzymatic double oxidation in organic media in 10 mL scale starting with biobased cyclohexanol. Results shown as a percentage in the crude reaction mixture, analyzed via GC.

	cyclohexanol / %	cyclohexanone / %	ε-caprolactone / %
	0	0	>99 %

After removal of the solvent *in vacuo*, ε-caprolactone was isolated with a yield of 64 % (72.8 mg, purity ~ 95 %).

^{1}H NMR (500 MHz, Chloroform-*d*) δ 4.25 – 4.19 (m, 2H), 2.69 – 2.58 (m, 2H), 1.81 (dtd, *J* = 48.8, 5.5, 3.0 Hz, 6H).

The data corresponds to the ones reported in literature.

Simulation of biotransformation on a semi-preparative scale without substrate

In order to identify the peaks in the high field of the ^{1}H-NMR spectra, the biotransformation was simulated on a semi-preparative scale without substrate (cyclohexanol). Subsequently, the reaction was processed in the same way as the biotransformations are typically processed in the MARS system. After removal of the solvent, a ^{1}H-NMR spectrum was measured from the remaining traces of the simulated biotransformation. In this spectrum, the same peaks can be detected in the high field as in previous biotransformations. It can be concluded that the peaks do not originate from by-products, but from extracted substances from the lyophilized cells. We attempted to eliminate these cell remnants *via* a syringe filter, however, this proved to be ineffective. Consequently, in the ultimate trial, we employed distillation for the work up of the next reaction, as this is a more prevalent industrial technique than purification via column chromatography.

For the simulation of the biotransformation, lyophilized cells (*E. coli* BL21(DE3)- pRSF-empty & pACYC-empty, 30 mg/mL, 300 mg) were introduced to *n*-butyl acetate (9 mL) in a Falcon tube, PPB (100 mM, pH 7, 1 mL) was added, and covered with oxygen. The reactions incubated for 6 hours at 25°C with 400 rpm shaking in a Falcon tube. Then 10 mL of ethyl acetate was added. The tube was vortexed, centrifuged at 20000 x g for 2 minutes, and then the

organic phase was isolated, and the solvent removed *in vacuo*. The remaining yellow solid was analyzed via ^{1}H-NMR-spectroscopy.

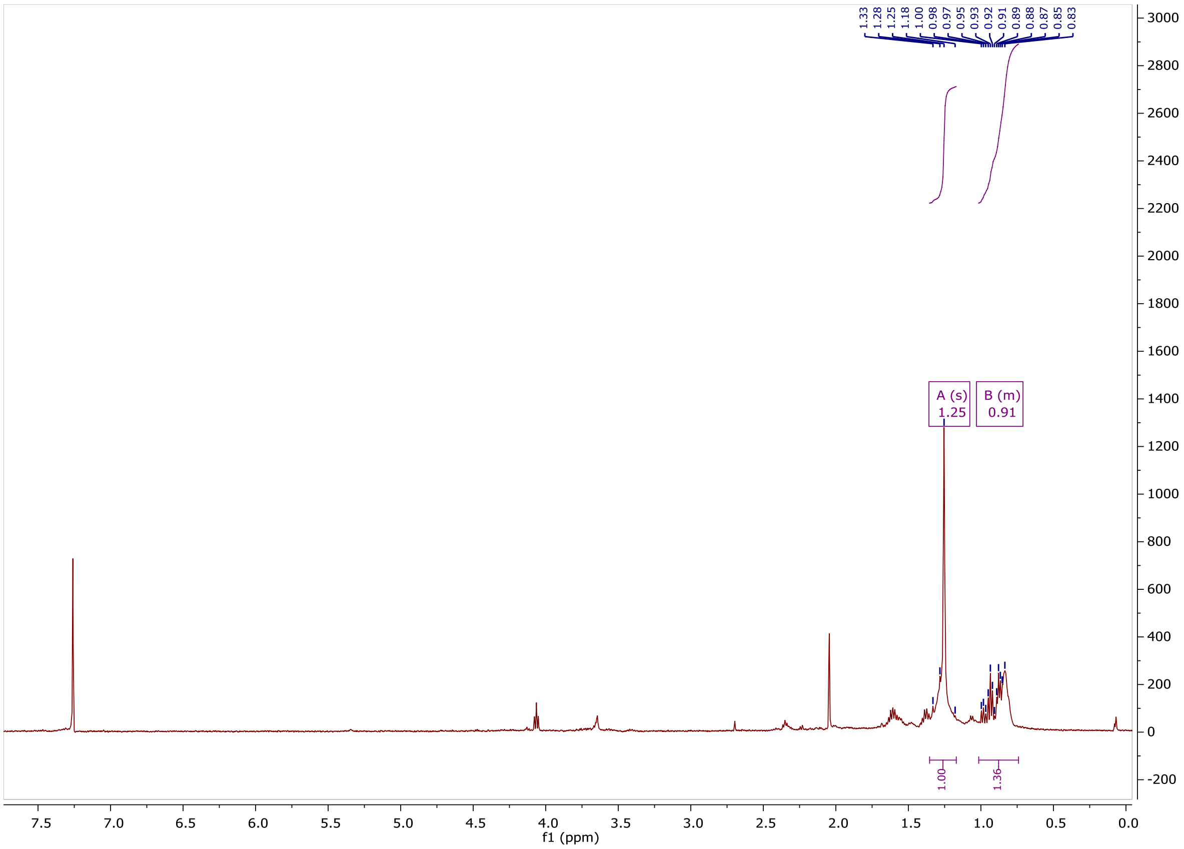

Figure S12: ^{1}H-NMR-spectrum of the simulation of the biotransformation.

Biotransformation in organic media - 100 mM conversion of bio-based cyclohexanol on a 100 mL preparative scale

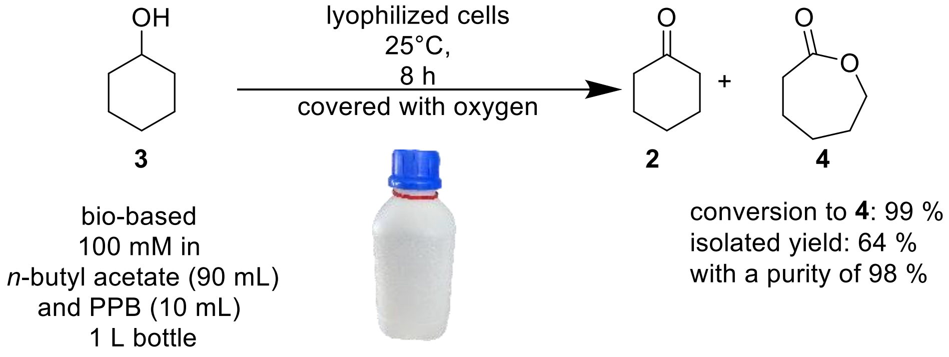

To achieve the double oxidation of bio-based cyclohexanol to ε-caprolactone, lyophilized cells (*E. coli* BL21(DE3)-pRSF-mAcinetoCHMO & pACYC-LkADH, 30 mg/mL, 3 g) were introduced to *n*-butyl acetate (90 mL) in a 1 L plastic bottle, PPB (100 mM, pH 7, 10 mL) and bio-based cyclohexanol (table S2, entry 5, 1 g, 100 mM) were added, and covered with oxygen. The reaction was incubated for 8 hours at 25°C with 400 rpm shaking in a plastic bottle. Then 100 mL of ethyl acetate was added. The bottle was vortexed, the suspension was centrifuged at 20000 x g for

2 minutes, and then the organic phase was isolated, dried over sodium sulfate and analyzed by gas chromatography.

Table S16: Results of the enzymatic double oxidation in organic media in 100 mL scale. Results shown as a percentage in the crude reaction mixture, analyzed via GC.

	cyclohexanol / %	cyclohexanone / %	ε-caprolactone / %
	0	1	99 %

After removal of the solvent by distillation *in vacuo*, ε-caprolactone was isolated with a yield of 64 % (726.5 mg, purity > 95 %).

^{1}H NMR (500 MHz, Chloroform-*d*) δ 4.24 – 4.17 (m, 2H), 2.68 – 2.56 (m, 2H), 1.90 – 1.68 (m, 6H).

^{13}C NMR (126 MHz, Chloroform-*d*) δ 176.31, 69.40, 34.67, 29.39, 29.07, 23.04.

The data corresponds to the ones reported in literature.[22]

5. NMR-Spectra and GC-Chromatograms

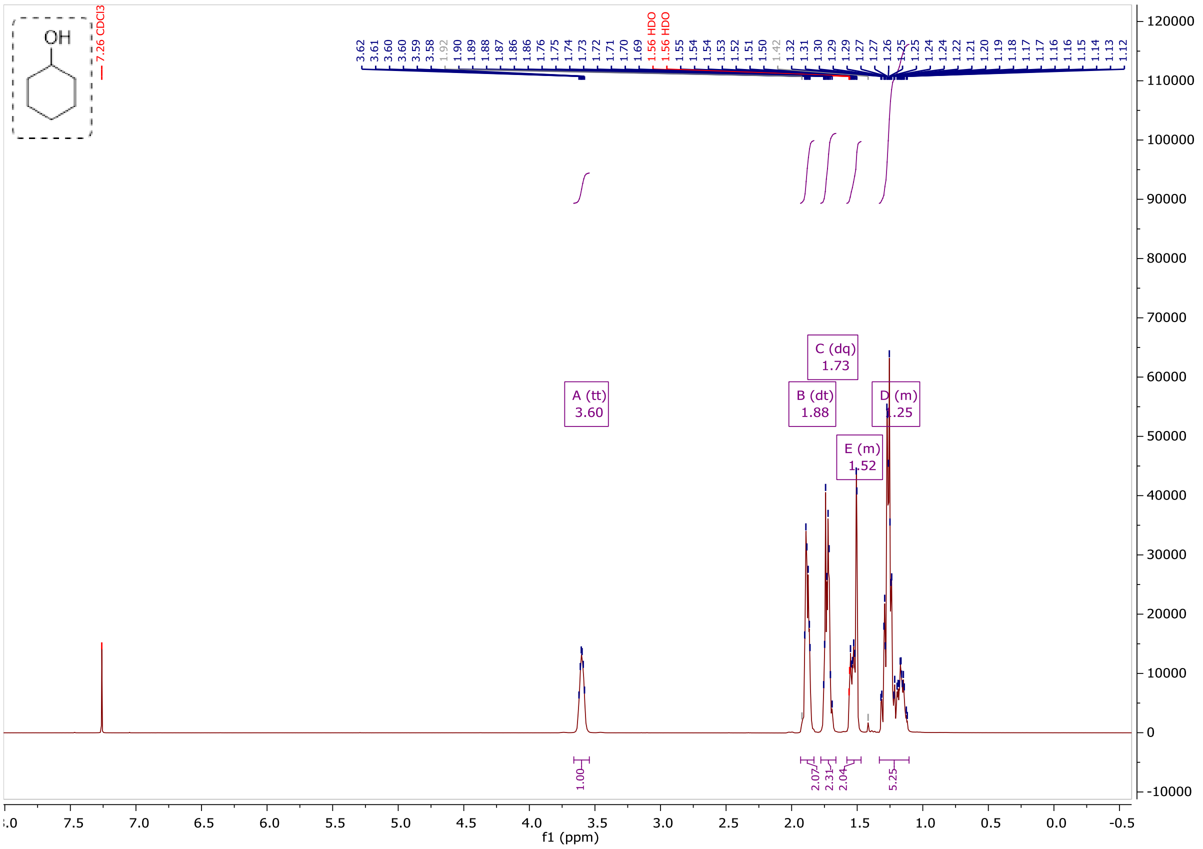

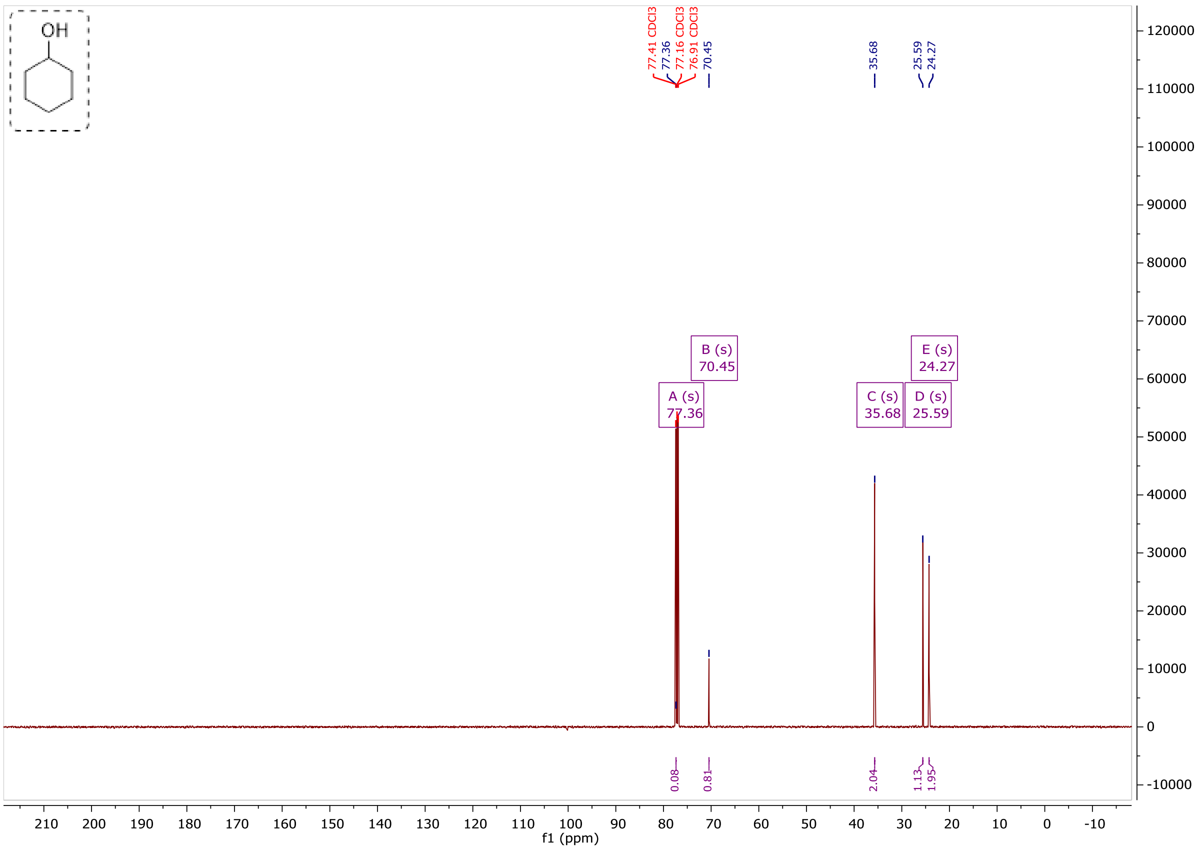
OH
77.41 CDCl3
77.36
77.16 CDCl3
76.91 CDCl3
70.45
35.68
25.59
24.27
A (s) 77.36
B (s) 70.45
C (s) 35.68
D (s) 25.59
E (s) 24.27
0.08
0.81
2.04
1.13
1.95
f1 (ppm)

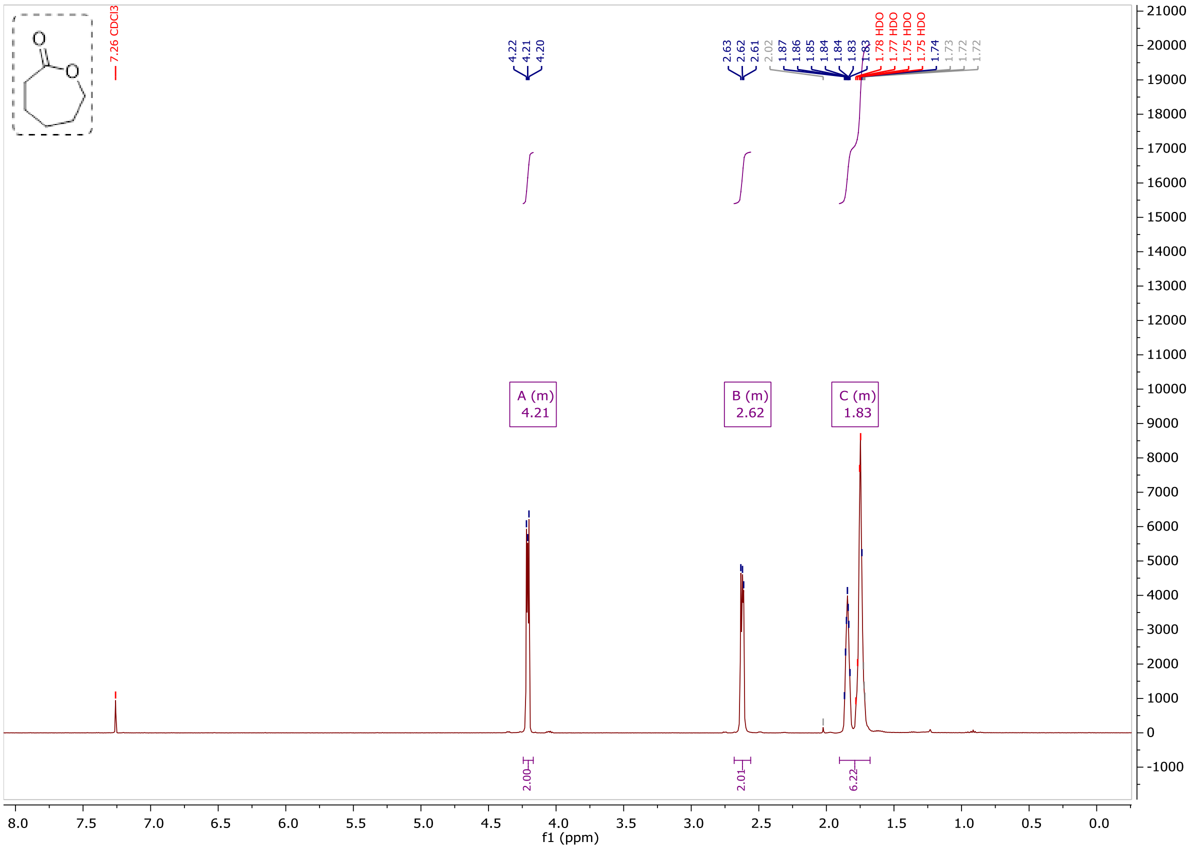
O
O
7.26 CDCl3
4.22
4.21
4.20
2.63
2.62
2.61
2.02
1.87
1.86
1.85
1.84
1.84
1.83
1.83
1.78 HDO
1.77 HDO
1.75 HDO
1.75 HDO
1.74
1.73
1.72
1.72
A (m) 4.21
B (m) 2.62
C (m) 1.83
2.00
2.01
6.22
f1 (ppm)

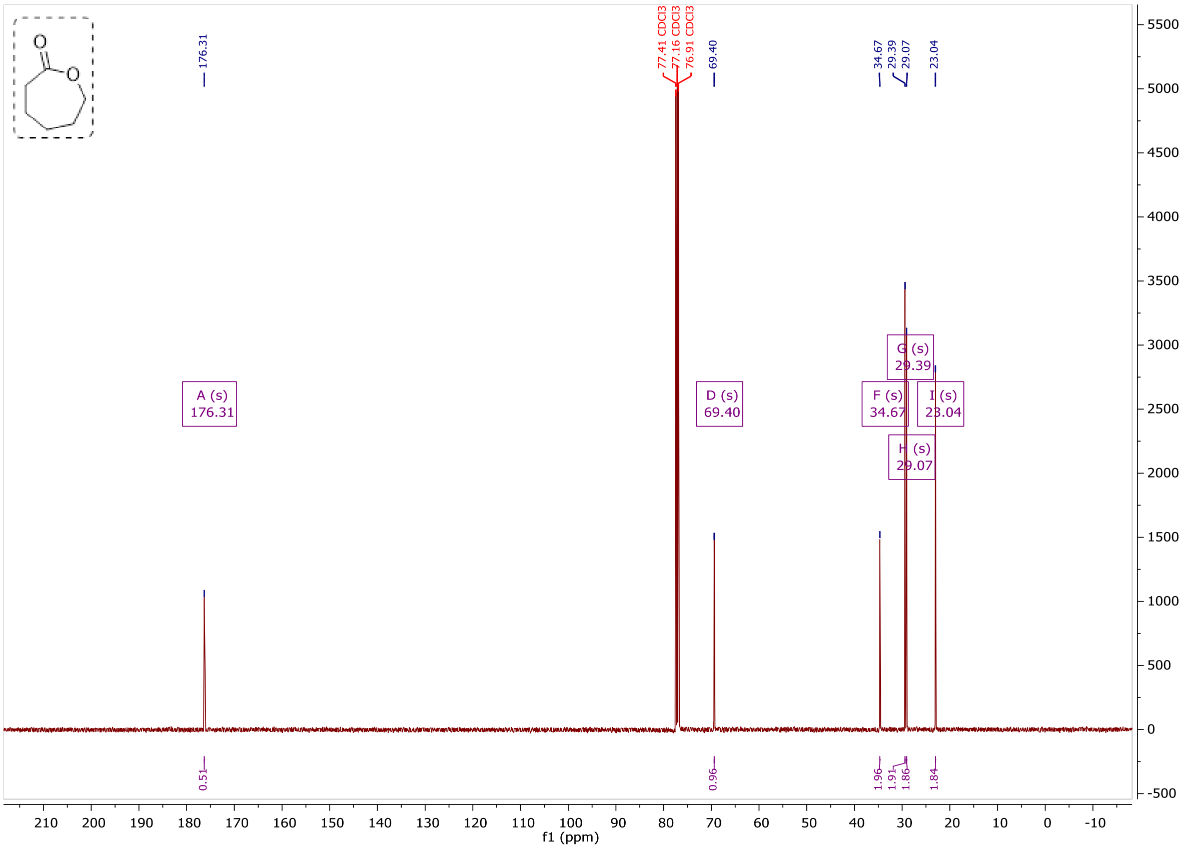

176.31
77.41 CDCl3
77.16 CDCl3
76.91 CDCl3
69.40
34.67
29.39
29.07
23.04
A (s)
176.31
D (s)
69.40
F (s)
34.67
G (s)
29.39
H (s)
29.07
I (s)
23.04
0.51
0.96
1.96
1.91
1.86
1.84
f1 (ppm)

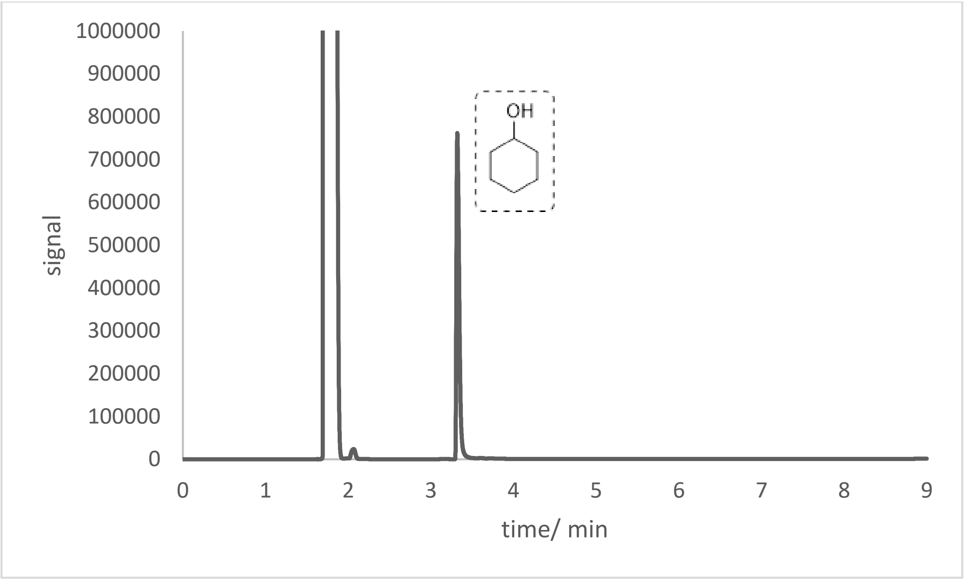

OH
signal
time/ min

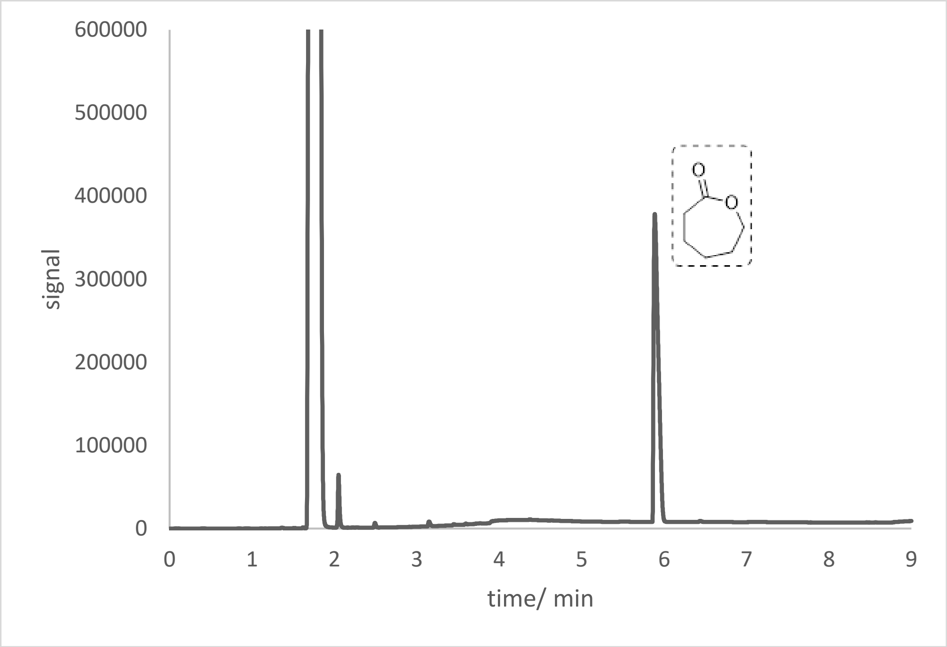

6. References

[22] S. Wedde, P. Rommelmann, C. Scherkus, S. Schmidt, U. T. Bornscheuer, A. Liese, H. Gröger, *Green Chem.* **2017**, *19*, 1286–1290.

[31] H. R. Mansouri, O. Gracia Carmona, J. Jodlbauer, L. Schweiger, M. J. Fink, E. Breslmayr, C. Laurent, S. Feroz, L. C. P Goncalves, D. V. Rial et al., *ACS Catal.* **2022**, *12*, 11761–11766.
